# Biochemical, Transcriptomic and Proteomic Analyses of Digestion in the Scorpion *Tityus serrulatus*: Insights into Function and Evolution of Digestion in an Ancient Arthropod

**DOI:** 10.1371/journal.pone.0123841

**Published:** 2015-04-15

**Authors:** Felipe J. Fuzita, Martijn W. H. Pinkse, José S. L. Patane, Maria A. Juliano, Peter D. E. M. Verhaert, Adriana R. Lopes

**Affiliations:** 1 Laboratory of Biochemistry and Biophysics, Instituto Butantan, São Paulo, Brazil; 2 Biotechnology Program, University of São Paulo, São Paulo, Brazil; 3 Laboratory of Analytical Biotechnology & Innovative Peptide Biology, Delft University of Technology, Delft, The Netherlands; 4 Department of Biochemistry, Institute of Chemistry, University of São Paulo, São Paulo, Brazil; 5 Department of Biophysics, UNIFESP, São Paulo, Brazil; University of North Carolina at Charlotte, UNITED STATES

## Abstract

Scorpions are among the oldest terrestrial arthropods and they have passed through small morphological changes during their evolutionary history on land. They are efficient predators capable of capturing and consuming large preys and due to envenomation these animals can become a human health challenge. Understanding the physiology of scorpions can not only lead to evolutionary insights but also is a crucial step in the development of control strategies. However, the digestive process in scorpions has been scarcely studied. In this work, we describe the combinatory use of next generation sequencing, proteomic analysis and biochemical assays in order to investigate the digestive process in the yellow scorpion *Tityus serrulatus*, mainly focusing in the initial protein digestion. The transcriptome generated database allowed the quantitative identification by mass spectrometry of different enzymes and proteins involved in digestion. All the results suggested that cysteine cathepsins play an important role in protein digestion. Two digestive cysteine cathepsins were isolated and characterized presenting acidic characteristics (pH optima and stability), zymogen conversion to the mature form after acidic activation and a cross-class inhibition by pepstatin. A more elucidative picture of the molecular mechanism of digestion in a scorpion was proposed based on our results from *Tityus serrulatus*. The midgut and midgut glands (MMG) are composed by secretory and digestive cells. In fasting animals, the secretory granules are ready for the next predation event, containing enzymes needed for alkaline extra-oral digestion which will compose the digestive fluid, such as trypsins, astacins and chitinase. The digestive vacuoles are filled with an acidic proteolytic cocktail to the intracellular digestion composed by cathepsins L, B, F, D and legumain. Other proteins as lipases, carbohydrases, ctenitoxins and a chitolectin with a perithrophin domain were also detected. Evolutionarily, a large gene duplication of cathepsin L occurred in Arachnida with the sequences from ticks being completely divergent from other arachnids probably due to the particular selective pressures over this group.

## Introduction

Scorpions are ancient arthropods which have the oldest known fossil record among the living arachnids dating from the Silurian period 428 Ma [1]. They are efficient predators presenting a varied diet (e.g., insects, spiders, solifugae, scorpions, isopods, gastropods, snakes, lizards, rodents) and it has been reported that scorpions can have their mass largely increased after one single meal [2] and survive up to one year starvation [3]. The hydrolysis of nutrients is achieved through a combination of extra-oral and intracellular digestion. Digestive enzymes are released by the secretory cells in prosomal midgut, anterior intestine and its respective digestive glands to be then regurgitated into the pre-oral cavity where the liquefaction of the chewed food starts. After being filtered by the coxapophyses, the liquefied nutrients will reach the prosomal midgut with the help of musculature from pharynx and esophagus. The predigested food is absorbed by pinocytosis and the intracellular digestion is performed inside the digestive cells from the midgut and midgut glands [4].

Prey capture and envenomation are well-studied processes since scorpionism is a world health problem [5] and also for the fact that the scorpion venom is a rich source of bioactive molecules [6,7]. However, few physiological processes related to digestion and digestive enzymes in scorpion species have been published. Sarin [8], Pavlovsky and Zarin [9] identified the first scorpion peptidases: pepsin, trypsin and chymosin. Said found cysteine catheptic activity in *Buthus quinquestriatus* [10]. Recent studies about digestive enzymes in scorpions have described the characterization of an amylase [11], a lipase [12] and a chymotrypsin from *Scorpio maurus* [13]. Due to all the presented characteristics, scorpions are particularly attractive animals for physiological and evolutionary studies, leading to the comprehension of evolutionary aspects of the feeding mechanism in Arachnida and Arthropoda and enabling the development of scorpion control strategies.

**Fig 1:**
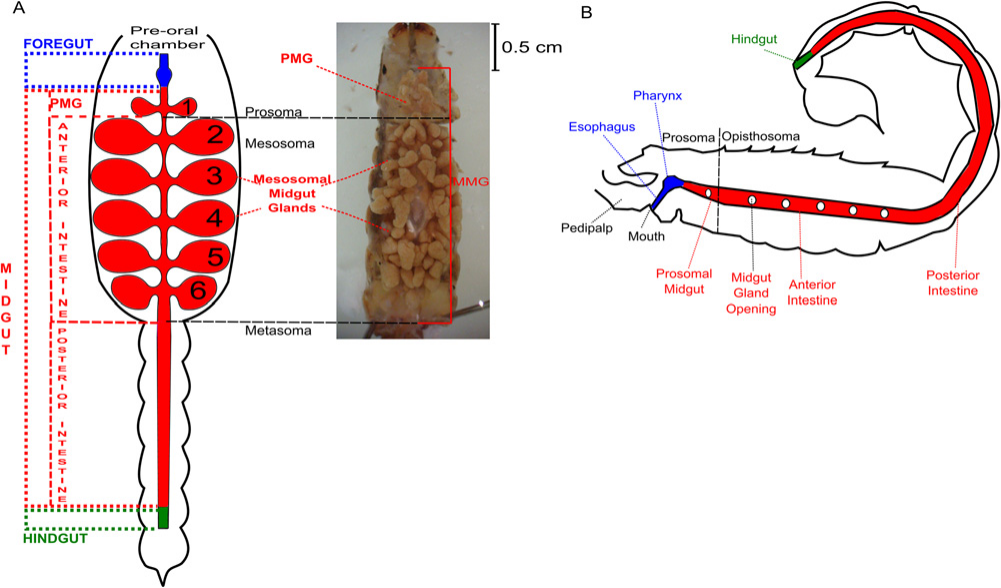
General morphology of scorpion digestive system and its location. Schematic ventral (A) and lateral (B) views of scorpion digestive system and its divisions. Right picture (A) represents ventral view of *Tityus serrulatus* MMG. PMG, prosomal midgut. Fig 1B was modified from [2].

At the onset of this study, neither DNA or complete protein sequence nor advanced techniques such as next generation sequencing and shotgun proteomics had been used to the investigation of scorpion digestive system (Fig 1). In this work, we investigated the molecular physiology of digestion in the scorpion *Tityus serrulatus* by using a combination of transcriptomic, proteomic and enzymological approaches, mainly focusing on protein digestion. A combination of transcriptomics and proteomics techniques together has previously been described as a strong approach in order to identify and to sequence DNA and proteins from non-sequenced organisms [14,15].

We here report a large dataset of transcribed and translated protein sequences in the midgut and midgut glands which we obtained from a scorpion. Quantitative proteomics and proteolytical data exhibited relative amounts and pH optima of different hydrolases. The main digestive peptidases were isolated and kinetically characterized. Subsequently, a phylogenetic analysis of cathepsin L and legumain was performed. For the first time, a more elucidative model for the digestive process in scorpions was proposed with evolutionary considerations about the feeding mechanism in Arachnida.

## Materials and Methods

### Animals and sample obtaining

Adult *Tityus serrulatus* females were obtained from the laboratory of arthropods at Instituto Butantan (São Paulo, Brazil). The animals were starved for at least 8 days and then fed with *Gryllus sp*.. After 9 hours of feeding, the animals still eating were separated from their prey and dissected whereas the starved ones were left without food for other 8 days before dissection.

After anesthetizing the animals in a CO_2_ chamber, the dissection was performed in a cold isotonic saline solution (300 mM KCl pH 7.0). The midgut (prosomal and anterior intestine), with its respective prosomal and mesosomal glands, are collectively referred as midgut and midgut glands (MMG) as a matter of simplification (Fig 1). Isolated MMGs from one specimen were homogenized with a Potter-Elvejhem homogenizer in ultrapure water (Milli Q) to 1 ml (final volume) and used as a sample source for the enzymatic assays and proteomic experiments. RNA extraction from MMG was achieved after dissection with sterilized instruments in autoclaved saline solution (300 mM KCl) containing 0.1% (v/v) diethyl pirocarbonate (DEPC).

### mRNA Library Preparation and Sequencing

All enzymes, primers and buffers cited in this section are from Illumina unless otherwise specified. RNA extraction was done using TRIzol reagent (Invitrogen) according the manufacturer instructions. The RNA amount was spectrophotometrically quantified at 260 nm and its purity evaluated by the absorbance ratio 260 nm and 280 nm. The RNA quality and integrity were analyzed in the Agilent 2100 Bioanalyser (Agilent Technologies).

Poly-adenylated mRNA was purified oligo(dT) magnetic beads (Illumina) according to their standard protocol (http:/grcf.jhmi.edu/hts/protocols/mRNA-Seq_SamplePrep_1004898_D.pdf). Thereafter, cDNA was reverse transcribed and cloned. In brief, the mRNA was fragmented in the proper buffer and the first cDNA strand synthesis was made using Superscript II Reverse Transcriptase (Invitrogen). After subsequent RNaseH treatment the second cDNA strand was synthesized by DNA polymerase I. The end of the molecules were phosphorylated and the 3´ terminal adenylated using the enzymes T4 PNK and Klenow exo, respectively. The adapters were then linked to the DNA fragments with a T4 DNA ligase. After that, the libraries were amplified with primers specific to the adapters.

The quality of the library constructed was validated by the Agilent 2100 Bioanalyzer (Agielnt Technologies) with the chip DNA 1000 and quantified by quantitative polymerase chain reaction with the kit KAPA Library Quantification (KAPA biosystems). The library was diluted to a final concentration of 20 pM and each one was clustered and amplified by using the TruSeq PE Cluster Kit v30cBot-HS. Next generation sequencing was performed in a HiScanSQ (Illumina) using the TruSeq SBS Kit v3-HS (200 cycles) according to the manufacturer’s instructions.

### Computational analysis

The HiScanSq (Illumina) data obtained were analyzed in four main steps. In the raw data obtainment step the software package CASAVA (2011) 1.8.2 (Illumina) was employed. This algorithm makes the base call from raw data transforming them into fastq format reads followed by the phred´s quality scores. The reads were visualized with the program FastQC 0.10.1 and then the Agalma pipeline shuffles the reads and removes those with low quality (less than 30 nucleotides). Next, vectors, primers and ribosomal RNA sequences were withdrawn after comparison with the Univec and ribosomal RNA databases, both from NCBI (National Center for Biotechnology Information).

*De novo* assembly was done by the programs Velvet/Oases incorporated to the Agalma pipeline [16,17]. Four assemblies were done to all samples with kmers of 31, 41, 51 and 61 that thereafter were merged and the redundant contigs removed. A BLAST (basic local alignment search tool- [18]) was used to identify and annotate assembled sequences using the UniProt as a database with an e-value threshold of 10^-10^. Fasta files were filtered by removal of transcripts smaller than 150 bp, splice variants and low confidence contigs. The final assembly of each physiological condition is available in S1 Dataset. Moreover, this transcriptome shotgun assembly project has been deposited at DDBL/EMBL/GenBank under the accession GBZU00000000. The version described in this paper is the first version GBZU01000000.

The gene ontology was obtained using the program Blast2GO [19] with the non-redundant NCBI database. The e-value and annotation cutoff were respectively 10^-6^ and 45. Subcellular location was predicted using the software WoLF PSORT [20]. The contig translation based on the DNA coding regions was performed using the software FrameDP v 1.2.0 [21]. After using the BLASTX tool against the UniProt database the program created a training set to predict the more likely coding DNA sequence (CDS) based on the interpolated Markov models (IMMs). Contigs with less than 50 amino acids were removed. The databases from fed and fasting animals were combined for the MASCOT searches (below) but the redundancy of the possible digestive enzymes was already manually removed by comparing the sequences. The database used for protein identification is available in data set S2. For the rest of the sequences the redundancy was removed using the program BLASTClust with sequence length coverage of 90% and a percent identity threshold of 97% after the MASCOT searches with the partially redundant database. This prevented discarding isoforms and partial sequences that contain an overlapping region but also different parts of the proteins.

### Proteomics procedures

The MMG homogenates of one specimen were submitted to three freeze and thaw cycles and then centrifuged for 20 min at 1,000 x g. Supernatants were collected and used for proteome analyses. Three distinct biological samples were individually separated by SDS-PAGE on a 10 well PAGE Novex 4–12% Bis-Tris Gel (Invitrogen, Bleiswijk, NL) for 30 min at a constant voltage of 200 V using MES-SDS as running buffer. Each gel lane was sliced in 32 equal pieces. Proteins were in-gel digested (trypsin) after reduction and alkylation, tryptic protein fragments were extracted from the gel with acetonitrile, vacuum dried and resuspended in 0.1 M acetic acid prior to analysis by nanoLC-MS/MS on an LTQ-Orbitrap Velos (Thermo Fisher) as previously described [22]. The raw LC-MS/MS data files were processed into peak lists using the software ReAdW 4.3.1. Mass spectra were deconvoluted using the program MS-deconv [23]. The files generated from MS-deconv were then analyzed by MASCOT (Matrix Sciences), an error tolerance of 0.05 Da was allowed only in the parent ion and also one miss cleavage site by trypsin. Data set S3 contains the peptide list of the entire proteome dataset and the software configuration used for the identification. Subsequently the MASCOT searches of all the runs were loaded together in the software Scaffold 4 [24] and statistically analyzed with X!Tandem [25]. Positive protein identification required the presence of at least 2 sequenced peptides with a false discovery rate (FDR) of 0.5%. Label-free quantitative analysis was done by normalized spectral counting using Scaffold 4. This is obtained by the sum of the spectral counting for each MS sample. They are then scaled so they are all the same and the scaling factor is applied to each protein. For quantification the biological replicates were separately analyzed and the protein relative abundance calculated for each sample. Due to this fact not all of the proteins identified in the general experiment appear in the list with quantifications.

### 2.5 Protein determination, hydrolase assays and peptidase classification

The protein concentration was determined according to Smith et al. [26] using egg albumin as standard. Peptidase fluorescent assays were performed using different substrates containing distinct fluorochromes and conditions (S1 Table). Fluorescence was measured with a Gemini Spectrofluorimeter (Molecular Devices) in their respective excitation and emission wavelengths. All assays were performed at 30°C and the measured activity was proportional to the protein concentration and the incubation time. No-enzyme and no-substrate controls were included. A combination of substrates, assay conditions and specific inhibitors were used to classify the peptidase activities at chromatographic fractions from MMG [27]. Inhibitors used were: 10 μM E-64 (cysteine peptidase), 10 μM CA-074, 10 μM pepstatin (aspartic peptidase), 1 mM PMSF (serine peptidase), and 5 mM benzamidine (serine peptidases). Chicken cystatin (0.5, 50 and 500 nM) from eggs (Calbiochem) was tested with the cysteine peptidase purified samples. In the assays with inhibitors, under either control or experimental conditions, the substrates were added after a 30 minute pre-incubation with the inhibitor at 30°C in the same buffers used for activity assays.

### 2.6 Isolation of cysteine peptidases

The samples from the homogenate of *Tityus serrulatus´* MMG containing 1 mM MMTS [28] were fractionated in 1.7 M ammonium sulfate for at least 16 hours at 4°C. The samples were centrifuged for 20 min at 16,100 × g and 4°C. The supernatant was applied to a hydrophobic column (Hitrap Butyl FF-GE) coupled to an ÄKTA-FPLC system (GE). Column was equilibrated in 50 mM phosphate buffer (pH 6) containing 1.7 M ammonium sulfate and eluted with a 25 ml gradient of 1.7–0 M ammonium sulfate in 50 mM phosphate buffer (pH 6); fractions of 1 ml were collected. Active fractions on Z-FR-MCA were pooled, desalted (HiTrap desalting column, GE) and concentrated using a Vivaspin 6 membrane (GE). The samples were then applied to a cation-exchange column (Resource S-GE) equilibrated in 50 mM sodium acetate buffer (pH 5). The protein was eluted using a 40 ml gradient of 0–0.6 M NaCl in the equilibrating buffer, and fractions of 0.5 ml were collected and assayed using Z-FR-MCA as described above. The two purified enzymes were visualized by SDS-PAGE and named cysp1 and cysp2.

### Acidic activation of cysteine peptidases

The crude MMG homogenate and the active pool after hydrophobic chromatography samples were diluted in 0.1 M citrate-phosphate buffer containing 3 mM cysteine and 3 mM EDTA at pH values ranging from 2.6 to 7.0 and incubated for 1 hour at 30°C. After that, samples were diluted in deionized water and the activity measured with 10 μM Z-FR-MCA in 0.1 M citrate-phosphate buffer (pH 5.5). The pH of these mixtures was checked. The condition with the highest rate of hydrolysis was selected and after that the homogenate was incubated for different periods of time in order to test the length of time that was required for acidic activation *in vitro*. After this incubation, enzymatic assays using Z-FR-MCA were performed as described above. Two controls were done: 1) the enzyme diluted in deionized water and incubated at 30°C for the same time as the activated enzymes or 2) the enzyme diluted in deionized water prior to the assay. The activity increase ratio was calculated as follow: activated sample activity/control. No differences between the two controls were observed thus the second one was chosen for the calculation. Standard activation of crude homogenate samples was performed by incubating the samples at 30°C at pH 2.6 for 1 hour.

### pH stability

The stability of the cysteine peptidases under different pH conditions was evaluated by incubating the activated enzyme samples from the MMG homogenates in buffers with different pH values at 30°C for 3 h or at -20°C for 24 h. The incubation buffers used were: 50 mM citrate-phosphate and 50 mM Tris-HCl. The samples were then 10 times diluted in deionized water to guarantee adequate pH for residual activity measurement. All buffers contained 3 mM cysteine and 3 mM EDTA.

### Effect of pH or substrate concentration on enzyme activity

The purified and partially purified samples described above were assayed with 10 μM Z-FR-MCA diluted in a series of 0.1 M citrate-phosphate buffers with pH values ranging from 2.6–7.0 and containing 3.0 mM cysteine and 3.0 mM EDTA. The effect of substrate concentration on the activity of the purified cysteine peptidases was studied using, at least, 15 different substrate concentrations (Z-FR-MCA and Abz-FRQ-EDDnp). The K_m_ values (mean ± SEM) were determined from a weighted linear regression using EnzFitter software (Biosoft). These assays were also performed in the presence of 5 different concentrations of pepstatin ranging from 1 to 50 μM.

The substrate Abz-FRQ-EDDnp was also completely hydrolyzed (16 hours at 30°C) by purified cysp 1 and cysp2 in order to confirm the cleavage site. The hydrolysis product was then applied to a C18 column (4.6 mm x 50 mm, Ace) coupled to an HPLC system (Shimadzu), and the products of interest were eluted using a linear gradient of 0–100% acetonitrile with 0.1% TFA as the polar solvent. The different fractions corresponding to the observed peaks were independently subjected to mass spectrometry using an MSQ-Surveyor instrument (Thermo) with electrospray ionization and the cleavage site was determined.

### 2.11 Phylogenetic analyses

A large set of metazoan cathepsins L (CTSL) and legumain (LEG) sequences obtained from public databases were used for the phylogenetic analyses. Alignments were conducted by the Muscle algorithm [29] with default parameters by using the MEGA v6.0 interface [30], with codons as anchors for the alignment. In some analyses, nucleotide positions with high entropy (i.e., high nucleotide substitution rates) were automatically trimmed using BMGE [31], to test if phylogenetic trees obtained with raw and trimmed alignments were significantly different, which would indicate alignment biases. Furthermore, regarding LEG, different portions were included in the final analyses: 1) the whole alignment; 2) without prepeptide; 3) without prepeptide and C-terminal; and 4) without prepeptide and C-terminal, but including GPI-transamidase (GPIt) sequences. Maximum likelihood (ML) including all complete and some of the incomplete endopeptidase sequences was done in IQTree v0.9.6 [32] using its ultrafast bootstrap method (set to 1,000 cycles), with data partitioning by codon position, with the best model for each position obtained from PartitionFinder v1.1.1 [33]. Concatenated Bayesian analysis (BA) was done in Beast v1.8.0 [34], with data partitioning by codon position, and assuming a lognormal distribution of evolutionary rates across branches in the topology (therefore we did not assume a strict molecular clock, but a relaxed one), by fixing its mean to 1.0 and letting the standard deviation follow an exponential (0.33) prior (program default). For each run, posterior probabilities of clades were obtained after discarding the burnin, which was assessed by graphical analysis in Tracer v1.6 [34]. The minimum number of gene duplication events were estimated in Notung v2.7 [35].

## Results

### Transcriptome and proteome general features

The data of *de novo* assembly results from the RNA-seq of the midgut and midgut glands (MMG) are summarized in S2 Table. About 30 and 36% of the contigs from fasting and fed animals presented BLASTX hits (S2 Table), respectively. After proceeding with the GO extraction 7,250 and 6,350 contigs of respectively fasting and fed animals were analyzed. The best BLAST hits results are related to the sequences of the tick *Ixodes scapularis* followed by other invertebrates (data not shown), which appears in accordance with phylogeny, as tick and scorpion both belong to Arachnida. The GO analysis related to the biological process, cellular component and molecular function of the transcriptomic data acquired identified sequences involved not only with the digestive process, but also in many different aspects of cellular homeostasis (S1 Fig). These results evidence that the deep mRNA sequencing performed was successful in retrieving a large number of gene products. Hence, the proteomic investigation was performed using the translated contigs as database.

The shotgun proteomics analysis retrieved a total of 845 proteins identified with at least 2 sequenced peptides and a false discovery rate (FDR) of 0.5% (S3 Table). Proteins identified in both conditions summed 553 sequences whereas 96 and 196 are exclusive to respectively fasting and fed animals (S3 Table). The GO from the identified proteins is exhibited in S2 Fig for fasting and fed scorpions. Sequences obtained in the proteomics analysis without BLAST hits summed 6.6 and 3% of all detected proteins in contrast to the 64 and 70% of unidentified contigs from MMG samples of fed and fasting animals, respectively.

### 3.2 Possible digestive enzymes identified in the transcriptome

A total of 238 different enzymes with a possible digestive role were found to be expressed in the MMG of the scorpion *Tityus serrulatus*. The different hydrolases sequences are distributed as follows: 32% exopeptidases, 31% carbohydrases, 20% lipases and 17% endopeptidases (Table 1).

**Table 1:** Possible digestive enzymes identified after the transcriptomic analysis in the midgut and midgut glands of the scorpion *Tityus serrulatus*.

Exopeptidases (32%)	Number of different transcripts	Carbohydrases (31%)	Number of different transcripts
Aminopeptidase N	2	Alpha-amylase	8
Xaa-Pro aminopeptidase	9	Beta-galactosidase	5
Methionine aminopeptidase	3	Alpha-glucosidase	8
Dipeptidyl aminopeptidase	4	Uncharacterized family 31 glucosidase	5
Glutamyl aminopeptidase	2	Mannosyl-oligosaccharide glucosidase	1
Aminopeptidase O	3	Chitinase	19
Leucyl-cystinil aminopeptidase	1	Alpha-L-fucosidase	4
Aminopeptidase NPEPL1	1	Mannosyl-oligosaccharide alpha-1,2-mannosidase	2
Carboxypeptidase N subunit 2	9	Alpha-mannosidase	21
Carboxypeptidase 1	3	**Lipases (21%)**	**Number of different transcripts**
Carboxypeptidase M	2	Monoacylglycerol lipase	4
Zinc carboxypeptidase A 1	1	Diacyglycerol lipase	7
Glutamate carboxypeptidase 2	1	Pancreatic-like triacylglycerol lipase	2
Carboxypeptidase Q	1	Gastric-like triacylglycerol lipase	2
Carboxypeptidase B	1	Pancreatic lipase-related protein 2	6
Carboxypeptidase D	1	Hormone-sensitive lipase	2
Carboxypeptidase E	1	Acid lipase	1
Lysosomal Pro-X carboxypeptidase	1	Patatin-like phospholipase	2
Dipeptidyl peptidase 10	2	Phospholipase A2	12
Dipeptidyl peptidase 9	1	Phospholipase B-like 2	4
Dipeptidyl peptidase 4	2	Phospholipase D1	4
Dipeptidyl peptidase 3	2	Phospholipase D3	1
Dipeptidyl peptidase 2	1	Other phospholipases	2
N-acetylated-alpha-linked acidic dipeptidase 2	15		
Xaa-Pro dipeptidase	2		
Alpha-aspartyl dipeptidase	1		
Tripeptidyl-peptidase 2	3		
**Endopeptidase (17%)**	**Number of different transcripts**		
Cathepsin L	11		
Cathepsin B	1		
Cathepsin F	1		
Cathepsin O	2		
Cathepsin D	2		
Legumain	1		
Astacin	14		
MAM domain-containing astacin	1		
MAM and CCP domains-containing astacin	1		
Zinc metallopetidase	1		
Chymotrypsin/Trypsin	6		

For the initial protein digestion all the four groups of peptidases were found to be represented. Metallopeptidases are the most abundant peptidase contigs with 17 sequences including 16 astacins and one zinc metallopeptidase. One of these astacins contains a MAM domain whereas in another one MAM and CCP domains are present. Cysteine peptidases are the second largest group with 16 sequences. Among then there are 11 cathepsins L, two cathepsins O, 1 legumain (TsLEG), 1 cathepsin B and 1 cathepsin F. Six serine peptidases with the catalytic residues from the trypsin family were found of which 3 contain the domains CUB and/or LDL. Finally also 2 cathepsins D-like aspartic peptidases contigs were identified. The number of different exopeptidases, with a total of 75 proteins, almost doubles the number of endopeptidases. Twenty six dipeptidases, 25 aminopeptidases, 21 carboxypeptidases and 3 tripeptidases were detected. Carbohydrases comprise 73 different molecules which are mainly constituted of chitinases (19 sequences) and alpha-mannosidases (21 sequences). The majority of lipolytic enzymes at the mRNA level are formed by 25 sequences of phospholipases but also monoacyl, diacyl- and triacylglycerol lipases were found with 4, 7 and 4 molecules each one, respectively.

### 3.3 Proteome data

#### 3.3.1 Quantitative and qualitative proteomics

A shotgun proteomics approach was applied in order to identify the proteins that are likely involved in the digestive process. A total of 844 proteins were identified and are displayed in S3 Table. Based on the sequences from the Table 1, the qualitative and quantitative data from the proteomics experiment are presented in S4 Table together with the scores for subcellular prediction using WoLF PSORT [20] and the presence or absence of the GO term for extracellular space and lysosome.

Label-free quantitative analysis using the normalized spectral counting of each experiment, showed a direct correlation with protein abundance [36]. Possible digestive enzymes comprise 6.2 ± 0.9% and 3.5 ± 0.4% of the identified proteins from the MMG of fasting and fed animals, respectively (S4 Table). In order to do an unbiased comparison of the digestive enzymes relative abundance in the MMG of fasting and fed scorpions, the data from S4 Table were used for a relative quantification considering the sum of digestive enzymes in each condition as 100% (S3 Fig). In the MMG of fasting animals the most abundant enzymes are chitinases which sum 46% of the digestive enzymes (S3 Fig). Chitinase 3 (10.8%) lacks the catalytic activity but it was included as a digestive protein since it may be involved in a peritrophic-like membrane/gel formation. After feeding a shift is observed with the most abundant post-feeding enzymes being cathepsin L1 (TsCTSL1), alpha-glucosidase and alpha-mannosidase (S3 Fig).

**Fig 2:**
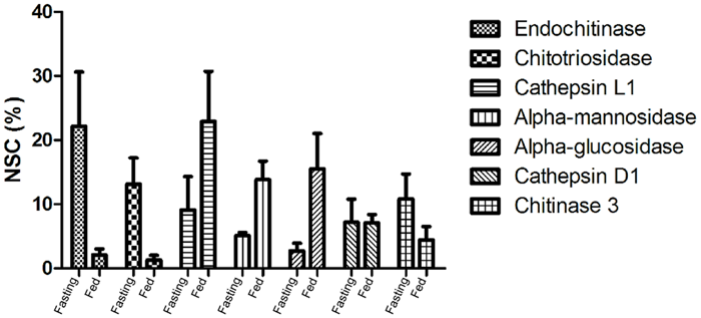
Quantitative analysis of selected proteins likely involved in digestion. Values are means and SEM from relative normalized spectra counting (NSC) calculated based on possible digestive enzymes identified. Shotgun proteomics experiment on triplicate samples with MMG of fasting and fed scorpions.

Fig 2 shows a comparison of some digestive enzymes identified in both physiological conditions. Once the number of replicates is low (n = 3) a statistical test was not applied. However, some trends can be observed based on the averages. For instance endochitinase, chitotriosidase and chitinase 3 are more abundant in the MMG of fasting animals, whereas TsCTSL1, alpha-mannosidase and alpha-glucosidase show the opposite trend (Fig 2). Cathepsin D1 seems to be constant in both conditions.

#### Subcellular prediction

The subcellular location of the possible digestive enzymes identified was performed by *in silico* analysis using the program WoLF PSORT [20]. S4 Table shows the scores calculated for the subcellular prediction. Additionally, sequence alignment and literature data were also used for the analysis and interpretation of *in silico* results.

Based on the prior knowledge that scorpions present extra-oral digestion combined with an intracellular phase [4] it can be assumed that digestive enzymes are the ones with extracellular and lysosomal signals. Databases on molecular localization prediction are mainly based on mammalian and yeast data and probably present few arachnid sequences. Thereby, even low k-NN values can be good evidences of protein location. GO terms from extracellular space and lysosomal sequences were used in order to corroborate WoLF PSORT data (S4 Table).

The lysosomal Pro-X carboxypeptidase had a high score for secretion and none for lysosome using WoLF PSORT analysis. However, in the GO analysis, the sequence was associated with lysosome. BLAST analysis of this sequence against the Uniprot database resulted in a high identity (e-value 1x10^-169^) with the known human lysosomal Pro-X carboxypeptidase. Thus, it is more likely that the scorpion enzyme is also inside lysosomes. These analysis indicated that the *in silico* prediction is just a first approach to digestive enzymes distribution which will have to be confirmed by imunocitolocalization studies.

All the complete endopeptidases identified by transcriptomic and proteomic analyses exhibited high *k*-NN values for extracellular location (S4 Table) and only TsLEG also had score for lysosome. TsLEG also had the lysosome GO term associated to its sequence and it was shown that in the tick *Ixodes ricinus* this endopeptidase acts inside the digestive vacuoles [37]. Hence TsLEG is probably a lysosomal enzyme as well. Cathepsin D was predicted as a secreted molecule by WoLF PSORT and as lysosomal by GO term. This enzyme is commonly associated with intracellular digestion [38] but it also can act extracellularly [39]. Ticks present intracellular cathepsin D activity with a digestive role and then, due to phylogenetic proximity, it is plausible that scorpion cathepsin D is also intracellular. CUB and LDL domains-containing trypsin 3 (TsCLTSP3) is likely secreted despite scores for other locations are also observed. Cathepsin F and cathepsin L2 (TsCTSL2) gave a score only for extracellular space, suggesting that these enzymes could be either secreted or lysosomal. Even though TsCTSL1 is incomplete at the N-terminal region, it is likely to be lysosomal on the basis of the arguments discussed below. Astacins 2 and 5a sequences are also incomplete. Nevertheless, these enzymes are normally active at alkaline pH and were found as secreted enzymes in the digestive juice of the spiders *Argiope aurantia* [40] and *Nephilengys cruentata* (Fuzita el al, unpublished). Hence we postulate that the astacins we detected in *Tityus serrulatus* are also secreted enzymes.

Endochitinase, chitinase 3, acidic chitinase, chitotriosidase and neutral alpha-glucosidase presented high signals for extracellular space in WoLF PSORT and also the GO term, so they are probably secreted enzymes. Lysosomal alpha-mannosidase and lysosomal alpha-glucosidase, as well as, beta-galactosidase 1 and 2 and beta-mannosidase are possibly lysosomal enzymes (BLAST identity analysis). Despite the small *k*-NN value for secretion and high value for endoplasmatic reticulum, alpha-amylase unlikely belong to this organelle and, presented the GO term for extracellular space as its supposed location. Spiders also employ secreted alpha-amylases as observed in *Nephilengys cruentata* (Fuzita et al, unpublished), *Tegenaria atrica* and *Cupiennius salei* [41].

The pancreatic lipase-related protein score for extracellular space is 25 and the GO term confirm the same location, indicating a possible secretion. Also lysosomal score was observed for this same enzyme and between all lipase sequences identified in this work after the RNA-seq this is the most similar with the N-terminal fragment of the purified digestive lipase from *Scorpio maurus* [12], with 54% identity and 61% similarity. In his study, this enzyme was found exclusively in the digestive vacuoles and not in the secretory granules [42], so it is plausible that this also is a lysosomal enzyme. Phospholipase B-like 2 is a lysosomal enzyme in humans [43] and it was mapped to the GO term lysosome, suggesting that it is a lysosomal enzyme.

### Enzymological approach

#### General features

**Fig 3:**
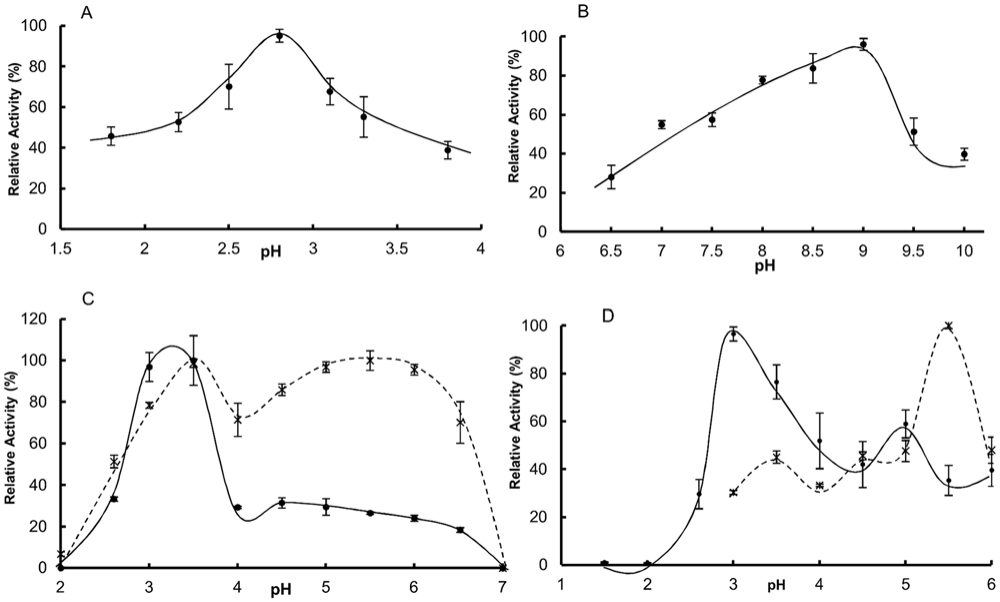
Effect of pH on endopeptidase activities using different substrates. Sample source was either crude MMG extracts (A and B) or chromatographically separated (C and D). (A) Hemoglobin 2%. (B) Casein-FITC 0.2%. C) Activated (✱) and non-activated (●) C1 samples. (D) Effect of pH on isolated cysp1 (✱) and cysp2 (●) samples. Buffers used (100 mM): Gly-HCl, pHs 1.5 and 2; Citrate-phosphate, pHs 2.6–7; MES, pH 7; TRIS-HCl, pHs 7.5–9; Gly-HCl 9.5–10. Buffers used in A, C and D contain 3 mM cysteine and 3 mM EDTA.

In order to investigate endopeptidasic activities involved in prey protein digestion, MMG homogenates of fed scorpions or chromatographically fractionated samples were tested with a series of substrates and inhibitors for cysteine, serine, aspartic and metallopeptidases under different assay conditions (S1 Table). Endopeptidases present in the scorpion MMG were able to cleave substrates in a pH ranging from 1.8 to 10, with peaks at 2.6–3.0, 5.5 and 8.0–9.0 (Fig 3 and Table 2). Although hemoglobin hydrolysis could be observed in very acidic pHs, the activity measured below pH 2 was highly unstable. Below the results are presented for each enzyme class separately.

**Table 2:** Peptidase absolute and specific activities involved in protein digestion in MMG from the scorpion *Tityus serrulatus* using different substrates.

Substrate; pH	Absolute Activity (U/MMG)	Specific Activity (U/mg)
Z-FR-MCA (3)	580 ± 80	16 ± 2
Z-FR-MCA (5.5)	700 ± 234	15 ± 5
Z-FR-MCA (8)	43 ± 2	1.2 ± 0.2
Z-RR-MCA (5.5)	81 ± 16	1.6 ± 0.2
N-Suc-AAPF-MCA (8)	4 ± 1	0.1 ± 0.04
Casein-FITC (8.5)**	2.1 ± 0.4	0.06 ± 0.03
Hemoglobin (2.8)**	28 ± 4	0.93 ± 0.04
Abz-FRQ-EDDnp (3)	35 ± 3	0.8 ± 0.2
Abz-FRQ-EDDnp (5.5)	2.2 ± 0.7	0.06 ± 0.02
Abz-GIVRAK-EDDnp (3)	0.42 ± 0.06	0.009 ± 0.001
Abz-GIVRAK-EDDnp (5.5)	0.16 ± 0.03	0.005 ± 0.001
Abz-GIVRPK-EDDnp (3)	0.18 ± 0.03	0.004 ± 0.001
Abz-GIVRPK-EDDnp (5.5)	0.2 ± 0.03	0.005 ± 0.001
Abz-GIVRAK-(Dnp)OH (3)	0.77 ± 0.06	0.018 ± 0.003
Abz-G-I-V-R-A-K-(Dnp)OH (5.5)	0.9 ± 0.3	0.025 ± 0.09
Abz-G-I-V-R-P-K-(Dnp)OH (3)	0.270 ± 0.004	0.006 ± 0.001
Abz-G-I-V-R-P-K-(Dnp)OH (5.5)	0.53 ± 0.04	0.015 ± 0.001
Abz-G-P-K-R-A-P-W-V-EDDnp (8)	0.9 ± 0.1	0.02 ± 0.004

Values are means and S.E.M of cleaved substrates in at least three different biological samples from *the* MMG of *Tityus serrulatus*. Assay conditions are listed in text.

#### Cysteine peptidases

**Fig 4:**
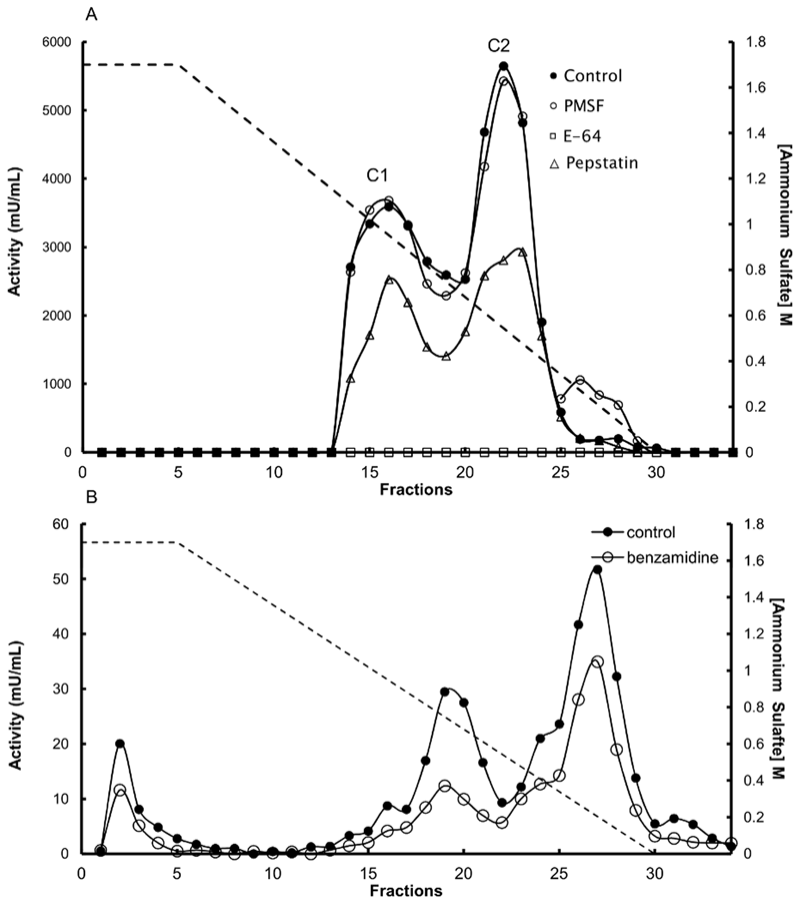
Hydrophobic chromatographic fractioning of *Tityus serrulatus* MMG homogenate. MMG homogenate was fractioned with 50% ammonium sulfate on a HiTrap Butyl column (GE) equilibrated in 50 mM phosphate buffer (pH 6.0). Elution was performed using a gradient of 1.7–0 M ammonium sulfate in the same buffer. (A) Activity of each fraction against 10 μM Z-FR-MCA was measured in 100 mM Tris-HCl buffer (pH 8.0) containing 10 mM CaCl_2_ (●) or in the presence of 5.0 mM benzamidine (○). (B) The activity of each fraction against 10 μM Z-FR-MCA was measured in 100 mM CP-buffer (pH 5.5) containing 3.0 mM cysteine and 3.0 mM EDTA in the absence (●) and presence of different peptidase inhibitors: (□) 10 μM E-64; (○) 1.0 mM PMSF; (Δ) 10 μM pepstatin.

The acidic Z-FR-MCA hydrolysis (pHs 3 and 5.5) has showed to be due to the action of cysteine peptidases after completely inhibition by E-64 (Fig 4A) and the need of cysteine in the assay buffer. Also, activity over hemoglobin (Fig 3A) was attributed to cysteine peptidases for the latter reason.

In ticks, hemoglobin hydrolysis by cysteine peptidases was already demonstrated [44,45]. The higher activities over Z-FR-MCA in acidic pHs rather than alkaline ones (Table 2) confirmed the previous observation of the quantitative data from S4 Table and S3 Fig, showing that cysteine endopeptidases (mainly cathepsins L1 and 2) are more abundant in contrast to serine endopeptidases. The cathepsin B identified in the transcriptomic experiment was only a 239 bp fragment, which could be the reason for the non-identification by mass spectrometry. Cathepsin B-like activity was observed using the substrates listed in Table 2 and S1 Table. However it is known that cathepsin B is also highly active over Z-FR-MCA [46]. All attempts to distinguish between cathepsin L and B activities resulted in only few clear interpretations due to the similarity between these both enzymes. By the usage of specific quenched fluorescent substrates (Table 2 and S1 Table), the comparison between Z-FR-MCA and Z-RR-MCA activities (Table 2) and CA-074 inhibition (data set S4), it seems that cathepsin L-like activity is higher than cathepsin B. The importance of cathepsin B is still unclear and needs further investigation. Legumain activity could not be detected.

#### Cysteine peptidases properties

**Fig 5:**
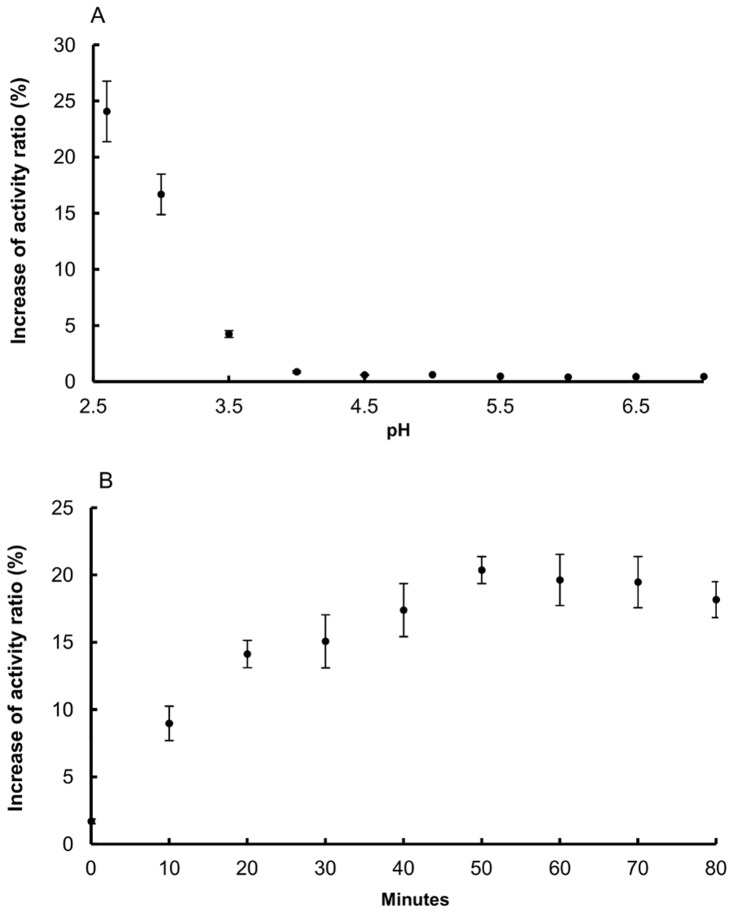
Acid activation of cysteine endopeptidases from *Tityus serrulatus´* MMG. Effect of incubating MMG homogenate (A) at 30°C for 60 minutes under different pH conditions. (B) Effect of time on acidic activation of cysteine peptidases from *Tityus serrulatus* MMG homogenate. After incubation in acidic buffer (pH 2.6), 2 μl of each enzyme preparation was assayed in 200 μl of 0.1 M CP buffer (pH 5.5) with Z-FR-MCA to measure activity at constant pH. Activity increase was calculated as ratio of incubated enzyme activity over non-incubated control activity. All buffers used for activation (0.1 M CP, pH 2.6–7.0) and activity assays contained 3.0 mM cysteine and 3.0 mM EDTA.

Due to the high activities over Z-FR-MCA this substrate was used for testing the cysteine peptidases properties in crude homogenate samples. An initial observation was that sample incubation in acidic pHs increased the activity over Z-FR-MCA. As, in general, cysteine peptidases are synthesized as zymogens [47,48], activation experiments under acidic conditions were performed. Fig 5A shows the activities of the crude homogenate samples after incubation for 1 hour at 30°C in solutions with different acidic up to neutral pH values. The hydrolysis of substrate was assayed as previously described in item 2.7 and no differences were observed in incubated or not incubated controls. Activation pattern was obtained after incubation at pH 2.6 (Fig 5A). Fig 5B shows the activation rate indicating that the maximal activity was obtained after at least 50 minutes of incubation at pH 2.6, 30°C. Loss of activity, most likely due to autolysis or pH instability, was observed only after 70 minutes of incubation. The same experiment was performed with partially purified samples in which the optimum pH for activation was 3 with an incubation time of 10 minutes at 30°C (data not shown). Thus, the standard activation procedure for crude homogenate samples was established as 60 minutes incubation at pH 2.6, 30°C. Activated and non-activated MMG homogenates submitted to gel filtration resulted in different elution patterns for the homogenate samples (S4 Fig). The non-activated samples exhibited two activity peaks, at 66 kDa and 44 kDa, independently of the substrate used. The activated samples exhibited only the 44 kDa activity peak, suggesting that the 66 kDa activity peak observed in the non-activated samples corresponds to the zymogen that was activated during the chromatographic process and/or acidic activity assay. The molecular mass differences between the active forms obtained using gel filtration (44 kDa) and electrophoresis may be a consequence of the different methodologies used.

The optimum pH over hemoglobin and Z-FR-MCA indicated that these enzymes present acidic characteristics (Fig 3A, 3C and 3D). We then tested the stability of the activated crude homogenate samples under a wide range of pHs after incubation at 30°C or -20°C. The enzymes presented a stability of approximately 100% between pH 3.0 and 6.5. At pH 8 or above the samples incubated at 30°C presented only 20% of the activity (S5 Fig). The cysteine peptidases in crude homogenate samples were highly thermal stable, 2 hours incubation of activated crude homogenate samples in temperatures up to 60°C did not result in activity loss (data not shown).

#### Purification of the cysteine peptidases

The following sequence of steps was used to purify two distinct cysteine peptidases: ammonium sulfate fractionation, hydrophobic chromatography and cation-exchange chromatography (S6 Fig). Two peaks of activity in the presence of Z-FR-MCA (C1 and C2) were observed during the hydrophobic separation (S6B Fig). When C1 was subjected to cation-exchange chromatography, two peaks of activity in the presence of Z-FR-MCA were observed (cysp1 and cysp2; S6C Fig). An SDS-PAGE analysis showed that both enzymes were effectively purified and exhibited molecular masses of 33 kDa (Figs 2 and 4D). The C2 fraction was also subjected to cation-exchange chromatography, but this fractionation did not successfully purify the enzymes. S5 Table shows the specific activity, yield and purification factor for each purification step at pH 5.5. Despite the (too) low recovery, this sequence of purification steps was the only one between different attempts by which a successful purification of the proteins was obtained.

The names cysp1 and cysp2 were kept since mass spectrometry experiments failed in sequencing the purified enzymes. However, after submitting C1 to identification by mass spectrometry three cysteine peptidases were identified (cathepsins L1, L2 and F). Cathepsins L1 and F are respectively the second and third more abundant identified proteins whereas TsCTSL2 is at the end of the list (S6 Table). It is likely that the purified cysp1 is cathepsin F and cysp2 is TsCTSL1. Cysp2 presented an optimum pH in 3 (Fig 3D) similar to the range 3–4 observed for the *Ixodes ricinus* cathepsin L1 [45]. Moreover Said observed an intracellular “cysteine catheptic” activity with optimum pH in 3 in the MMG of the scorpion *Buthus quinquestriatus* [10]. So far, none described cathepsin F presented such optimum pH range. Cysp1 optimum pH was 5.5 (Fig 3D) and it seems that this enzyme is one of the zymogens present in the sample. Activation experiments followed by optimum pH profile with C1 as enzyme source showed that without activation only about 26% of the activity can be observed at pH 5.5 (Fig 3C). After acidic activation a new peak is observed at pH 5.5 (Fig 3C), which is the same optimum pH of the purified cysp1.

Both purified cysteine peptidases cleaved the substrate Abz-FRQ-EDDnp with the Phe at P2 position and were not able to cleave Z-RR-MCA. The K_m_ values (S7 Table) that were obtained with Z-FR-MCA were 8.4 and 45 μM for cysp1 and cysp2, respectively, whereas K_m_ values of 0.02 and 0.06 μM were obtained when Abz-FRQ-EDDnp was used as substrate. The V_max_/K_m_ ratios that were determined with Z-FR-MCA were 390 for cysp1 and 13 for cysp2, whereas the V_max_/K_m_ ratios determined when Abz-FRQ-EDDnp was used were 3790 and 660 (min^-1^). These values indicate that cysp1 is catalytically more efficient than cysp2. Analysis of S2 subsite from the three identified cysteine peptidases in C1 fractions showed different amino acid composition, indicating a different binding affinity for each enzyme.

#### Pepstatin inhibition in family C1 (clan CA)

Classification assays using combinations of different substrates and inhibitors indicated that enzymes present in the chromatographic pool C1 could be inhibited by pepstatin (Fig 4A). Purified samples of cysp1 and cysp2 were assayed in the presence of different pepstatin concentrations. The resulting Lineweaver-Burk plots are shown in S7A Fig. The lines in these plots intersect the x-axis to the left of the origin as the pepstatin concentration increases, indicating that the K_mapp_ values increase with higher pepstatin concentrations. The V_max_ values were equal to the control values when 1, 5 or 10 μM pepstatin was used. Nevertheless, the addition of 25 or 50 μM pepstatin resulted in a decrease in V_max_, which can be observed as the lines crossing the y-axis at higher values (S7A Fig). A replot of the reciprocal plot versus the corresponding inhibitor concentration (S7B Fig) shows that pepstatin is a competitive inhibitor [49] of cysp1 with a K_i_ of 40 μM. Cysp2 was also inhibited by pepstatin, but the experiments did not provide a clear pattern for the inhibition in this case.

Although pepstatin is a tight binding inhibitor of aspartic peptidases with a K_i_ of 45 pM [50] some cysteine peptidases, calpains (clan CA, family C2) [51] and legumains (clan CD, family C13) [52] are inhibited by pepstatin. Apparently, up to 10 **μ**M, pepstatin inhibits cysp1 via a reversible competitive mechanism, with a K_i_ of 40 **μ**M (S7B Fig). Cysp2 is also inhibited by pepstatin; however, it was not possible to determine the mechanism of this inhibition (data not shown). A reason for this competitive inhibition is the higher magnitude of the calculated K_i_ (40 **μ**M) for cysp1 is contrast to the cathepsin D K_i_ (45pM). Nevertheless, the recommended use of pepstatin is in the micro molar range when screening for peptidase activity [27] and till now such kind of inhibition was not reported for C1 family.

#### Serine endopeptidases

The alkaline hydrolysis of casein-FITC suggested the presence of serine and metallopeptidases. The former was corroborated by hydrolysis of Z-FR-MCA and N-Suc-AAPF-MCA at pH 8.0 (Table 2) and identification by mass spectrometry (S3 Table and S3 Fig). Activity over Z-FR-MCA was higher than N-Suc-AAPF-MCA (Table 2) indicating more participation of trypsin-like enzymes in contrast to chymotrypsin in the digestive process. This activity is calcium dependent; no activity was observed in homogenate samples dialyzed against EDTA in the absence of CaCl_2_, while the absolute and specific activities were recovered in the presence of 10 mM CaCl_2_. Subsequently, the activities of chromatographic fractions against Z-FR-MCA at pH 8 were tested in the presence of a trypsin inhibitor. The hydrolysis of Z-FR-MCA was inhibited by at least 45% in the presence of benzamidine at pH 8 (Fig 4B). Thus, the enzymological results showed trypsin and chymotrypsin-like activities. Nevertheless, no correlation with direct protein analysis was obtained. The only serine endopeptidase identified by proteomics, TsCLTSP3, does not allow to make such correlation without further investigation.

#### Aspartic and metalloendopeptidases

The activity of astacin-like metallopeptidases identified by mass spectrometry could not be clearly distinguished from the serine peptidase activities. The observed activities of crude homogenate samples on casein-FITC and Abz-GPKRAPWV-EDDnp seem to be result of a mixture of distinct enzymes such as metallo- and serine peptidase (Table 2). Activity assays using casein-FITC after chromatographic separations in the presence of inhibitors presented too low activity (data not shown) to draw any conclusion.

The hydrolysis of hemoglobin under acidic conditions indicated the presence of aspartic and cysteine peptidases. Both types of enzymes were indeed detected by our mass spectrometry analyses (S3 Table). However, the hydrolysis of hemoglobin was completely dependent of cysteine and EDTA presence in the assay medium. In addition to that, the absence of hydrolysis of an aspartic peptidase substrate (Table 2) corroborates that, probably, hemoglobin hydrolysis is dependent on cysteine peptidases. Hence, it was not possible determine aspartic peptidase activity in MMG samples of *Tityus serrulatus*.

### Other molecules identified in the midgut and midgut glands

Regardless of the molecules related to organism homeostasis and the possible digestive enzymes above described, some proteins that are indirectly associated with digestion were also identified at the protein level. Proteins related to the vesicular trafficking such as clathrin (light and heavy chains), Rab (1a, 2, 5c, 11a and 14), sorting nexin (2, 6, 12 and 17) and proteins related to vesicular acidification (V-type proton ATPase subunits A and B) could be detected. Two MAM and LDL-receptor class A domain-containing were identified in the MMG probably related to endocytosis. Peptidase inhibitors like cystatin and serpin as well as one beta-galactosidase activator (lysosomal protective protein) were also present. Moreover, 3 different toxins (U_24_-ctenitoxin-Pn1a) with similarity to cysteine peptidase inhibitors from the venom of the spider *Phoneutria nigriventer* were found transcribed and translated in the midgut glands of the scorpion *Tityus serrulatus*. This is the first report of such toxin expressed and translated in the digestive system of a scorpion.

### Phylogenetic analyses

#### General considerations

**Fig 6:**
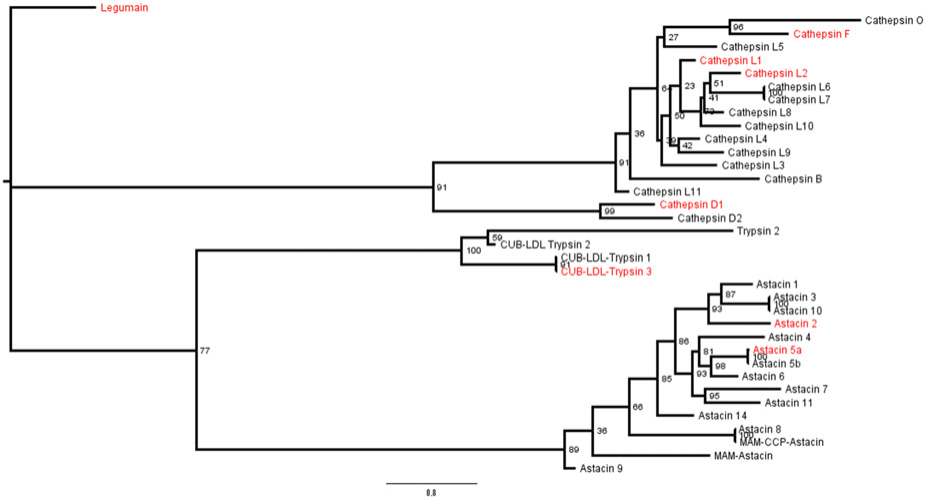
Phylogenetic relationships among endopeptidases present in MMG of the scorpion *Tityus serrulatus* using maximum likelihood algorithm. Sequences displayed in red were identified by proteomics.

All complete and some of the incomplete endopeptidase sequences were used to infer a ML and BA phylogenetic trees. Similar results were obtained with both algorithms so we decided to use the ML analysis (Fig 6). The cysteine peptidases of family C1A formed a monophyletic group comprising cathepsins B, L, F and O. Cathepsins F and O are the closest related, whereas cathepsins B and L11 are more divergent. Interestingly, cathepsin D formed a sister group to the remaining cysteine cathepsins with a bootstrap value of 91% (Fig 6), which was also observed with high posterior probability using BA (data not shown). TsLEG, as expected, is an isolated branch in the tree. Trypsins and astacins form separated monophyletic groups, but their relationship as sister groups is not strongly supported.

#### Cathepsin L and legumain evolution in Metazoa

**Fig 7:**
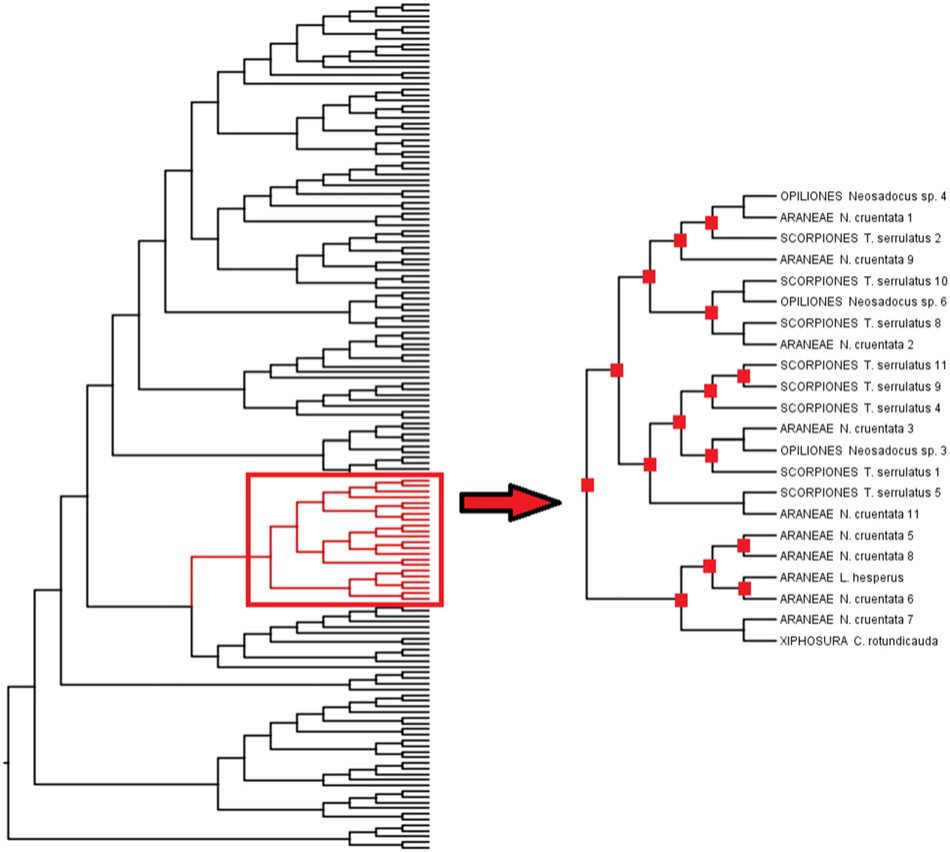
Section of Cathepsin-L Bayesian phylogeny including only clade Arachnida 3. For whole topology see S8 Fig Red squares indicate duplications (15 in total) as inferred by Notung v2.7.

Probably due to positive selection on biochemical properties, saturation of the phylogenetic signal throughout the time spanned by the evolution of the organisms analyzed, and also to possible inclusion of paralogous sequences, the CTSL alignments posed some difficulties in retrieving known phylogenetic relationships among taxa. Nevertheless, four different duplication events were detected in metazoans (S8 Fig). In the first duplication event, TsCTSL3 is in a group with papain, which does not include deuterostomes. Regarding arachnids, we obtained the groups named Arachnida 1, 2 and 3. Arachnida 1 is almost exclusively formed by Parasitiformes but two sequences from Opiliones can also be found in this group. Arachnida 2 is formed by an ortholog present in Acariformes, Araneae, Scorpiones e Opiliones, indicating that this gene was present in the ancestor of arachnids. Fifteen further duplications could also be detected within Arachnida 3 (Fig 7).

The TsLEG has a unique feature among known sequences of metazoans. As the legumain-like enzymes from prokaryotes it lacks the C-terminal extension (C-term). Shutov et al [53] proposed that the ancestral legumain sequence would be shorter than the derived ones since the C-term is not necessary for activity [54]. Although they presented a preliminary neighbor joining tree, a broader phylogenetic analysis including metazoan legumains was still lacking, so we carried it for the present study. We have performed both ML and BA with and without each of the prepeptide, C-term, and GPI-transamidase (GPIt) sequences, in all cases using the raw alignment, or else an alignment with highly polymorphic sites removed. The scorpion sequence behaved as a ghost taxon (i.e., changing its position in the phylogeny in different analyses), but excluding it before the analyses did not improve the remaining taxon relationships. All these analyses indicated similar trees, with overall higher support when including GPIt.

## Discussion

### Digestion in scorpions: cellular, enzymatic and compartimentalization aspects

The works of Said [10], Goyffon and Martoja [4] and Zouari [12,42] gathered, until now, the main information about the digestive process in scorpions. They suggested that the first step of digestion, the quick prey digestion, occurs extracellularly and; the final digestion may occur intracellularly. Goyffon and Martoja identified the two main cellular types involved in prey digestion and the secretion granules produced before a prey capture, in which, they assumed, the digestive enzymes reside. Said identified some proteolytic activity suggesting the participation of these enzymes in digestion and Zouari evidenced an intracellular lipase.

By combination of different methodologies including enzymological assays, two high throughput techniques (next generation sequencing and shotgun proteomics) and bioinformatic tools we have identified 238 proteins (Table 1) likely involved in catabolism of nutrients and the organization of the digestive process in the scorpion *Tityus serrulatus*. Of these 43 were identified at the protein level (S4 Table). The gene ontology (S1 and S2 Figs) evidenced that the combination of these two high throughput techniques, is very efficient to do a *de novo* assembly of the proteins from an organism with an unsequenced genome. Besides that, some phylogenetic assumptions can be deduced from a comparative analysis of available sequences in public databases. Furthermore, these data allowed the corroboration of the histological data described by Goyffon and Martoja that unfed scorpions already presented all proteins involved in the extracellular phase of digestion. However, there could be a limitation to the use of these technologies, mainly regarding the fed animals, due to contamination of mRNA and proteins from the prey. It is not possible to avoid food contamination since it is inherent to the feeding process and to the morphological characteristic. However, contamination would only be observed in fed animals. The analyses performed with fasting scorpions avoided this kind of contamination and many of the identified digestive enzymes could be found in both conditions. Although the RNA-seq is a sensitive technique there is a large proportion of MMG tissue in contrast to the partially digested prey, even assuming that non-degraded mRNA of the prey could be found at this point and sequenced. Moreover, the main BLAST hits obtained were related to the tick *Ixodes scapularis*, showing the similarity of our data set with another arachnid. Another example is the phylogenetic analysis performed with the identified cathepsins L, in which none of identified *Tityus serrulatus* enzymes grouped better with insects rather than arachnids. In conclusion, if there is contamination, we think that it is not significant in face of the obtained results.

Regarding protein digestion in scorpions, we obtained transcriptomic and proteomic evidence for all four main endopeptidases usually involved in protein hydrolysis which comprises distinct enzymes covering a range of pH from very acidic to alkaline medium. This suggests that digestion should occur in at least two distinct compartments with distinct pH conditions. The hypothesis of an alkaline extracellular phase of digestion has already being proposed on the observations regarding the pH of action of enzymes found in the digestive juice of spiders [40,55,56]. In contrast to that, an acidic intracellular phase of digestion is well characterized in ticks [38,57]. The enzymological data together with the differential quantitative analysis and the subcellular prediction used, in general, also supported this hypothesis. Subcellular prediction tools allowed some inferences about which enzymes seem to be involved in the extra and intracellular phases of digestion. Cysteine peptidases (cathepsins B, F, L1, L2 and legumain) and the aspartic peptidase cathepsin D1 are likely responsible for the acidic digestion in the digestive vacuoles, in contrast to astacins (2 and 5a) and TsCLTSP3, which probably hydrolyze peptide bonds extracellularly under alkaline conditions. Besides that, exopeptidases like Pro-Xcarboxypeptidases and carboxypeptidase Q, exoglycosidases as alpha-mannosidase, beta-mannosidase, alpha-glucosidase, beta-galactosidases and phospholipase B and pancreatic lipase related-protein were detected as additional components of the digestive vacuoles. On the other hand, secretory granules responsible for the extra-oral digestion seem to contain: chitinases, alpha-amylase, alpha-glucosidase, pancreatic lipase related-protein, alpha-fucosidase, besides astacins and trypsins.

The chitinolytic activities, as secreted enzymes and compounding, possibly, a digestive juice, make the scorpions efficient insect predators. Among chitinase sequences, we identified a chitolectin (chitinase 3) with a peritrophin domain which is catalytically inactive, since it does not present the catalytic residues. It is the first time that the presence of a peritrophic gel/ membrane is suggested in a scorpion. However, there is some evidence for this structure in Arachnida [58–60]. The function of this peritrophic structure is still unknown in Arachnida, but in Insecta and Crustacea it has a compartmentalization function in the digestive process [61,62]. The obtainment of all these sequences will allow their expression in heterologous system and the confirmation of their location by immunohistochemistry analysis.

### Digestive peptidases

#### Acidic protein digestion

The cysteine peptidases from the scorpion MMG are active only at acidic pHs (Figs 3 and 5, and Table 2). All together, these enzymes are the most abundant class of peptidases summing about 30% of the digestive enzymes in fed Tityus serrulatus´ MMG (S3 Fig), which is a strong evidence of their importance in the digestive process. CTSL has confirmed to be quantitatively the most important endopeptidase for the initial protein digestion by activity assays (Table 2) and quantitative mass spectrometry (S3 Fig). Twelve different genes coding for cathepsin L were identified and 2 could be confirmed by mass spectrometry (Table 1 and S3 Table). The highest activities were observed using Z-FR-MCA under acidic conditions (Table 2) and also, in the quantitative proteomic analysis, cathepsins L1 and 2 sum 11 and 27% of the digestive enzymes in the MMG of fasting and fed animals, respectively. Moreover, it seems that feeding causes an increase in TsCTSL1 abundance as shown in Fig 2. In the best studied arachnid group, the Parasitiformes, CTSL has already been shown to be an important digestive enzyme [38,63,64]. However, this is the first study to clearly demonstrate such importance in the digestive process of a predator arachnid. Other cysteine peptidases were also detected such as cathepsin B, F and legumain. TsLEG and its mRNA could be found solely in the MMG of fed animals, indicating a correlation with the feeding stimulus. Based on the literature data about the use of legumains in the digestive process of ticks [37,52,65], it is possible that also in scorpions this enzyme is involved in either prey´s protein degradation and/or trans-activation of clans CA and AA endopeptidases.

Cathepsin F presents similarities as pH of stability and optimum pH similar to CTSL [66]. In humans, it is associated with antigen processing and presentation [67] and, recently, it has been reported as part of a multidomain gene in the arthropod *Manduca sexta* [68], but its role in this insect could not be determined yet. In parasitic helminthes this cysteine peptidase can be secreted outside its body [69,70] and/or be expressed in the gut participating in the host ´s hemoglobin degradation [71,72]. In *Tityus serrulatus* this enzyme presented the cystatin domain in the propeptide region and the same optimum pH 5.5 as human and *Clonorchis sinensis*´ cathepsin F [66,71] which is slightly more acidic than other helminthes ones [73,74]. The scorpion cathepsin F is probably involved in food digestion, though other roles as trans-activation of other peptidases cannot be discarded. To our knowledge, this is the first report of such enzyme expressed and translated in the midgut of an arthropod.

Besides cysteine peptidases, aspartic peptidase as cathepsin D1 was identified at the mRNA (Table 1) and proteomic levels (S3 Table) although its activity could not be detected using typical synthetic substrates. This cathepsin D1 is the second most abundant peptidase after TsCTSL1, quantitatively corresponding to about 7% of the digestive enzymes in either fed or fasting animals (S3 Fig). This constancy could be evidence that cathepsin D1 will be increased after 9 hours of feeding or that it will not be affected by feeding stimulus and it is a constitutive enzyme. In ticks, this enzyme plays an important role in the acidic proteolysis performed inside the digestive cells. However, it is quantitatively less abundant than cathepsins B, C and legumain [38]. In contrast to that, other arthropods secrete cathepsin D to perform luminal digestion [39].

#### Alkaline protein digestion

**Fig 8:**
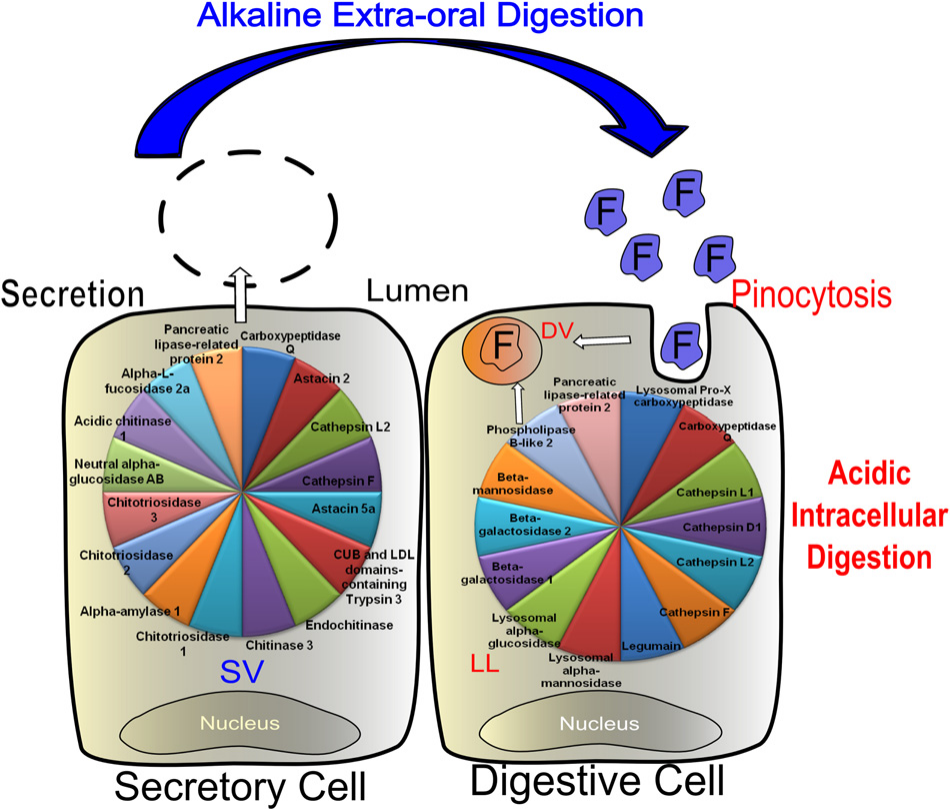
Schematic representation of midgut and midgut glands secretory (SC) and digestive cells (DC). Figure displays enzymes present in secretory vesicles (SV) and lysosome-like (LL) organelles. Lysosomes probably fuse or exchange contents with pinocytic vesicles to end up in digestive vacuoles. DC: digestive cells, DV: digestive vacuoles, F: pre digested food, M: mitochondria, P: pinocytosis, RER: rough endoplasmic reticulum, S: spherites, SC: secretory cells.

Alkaline proteolytic activity in *Tityus serrulatus* is probably the result of the sum of metalo- and serine peptidases. TsCLTSP3 presented a strong score for secretion using prediction software (S4 Table), which is an indicative of the alkaline extracellular/extra-oral digestion performed by scorpions (Fig 8). Quantitatively, these enzymes are less abundant in contrast to the cysteine peptidases. This observation comes not only from the activity assays (Table 2) but also from the quantitative proteomics (S3 Fig). Curiously, none of the complete trypsin-like sequences are composed exclusively by the trypsin domain as is tipically observed in insect [75] and digestive vertebrate trypsins. The sequences of the identified trypsins always contain a CUB domain and, sometimes, also a LDL domain. The presence of the latter domain in TsCLTSP3 is one possible explanation for the calcium-dependent activity observed in the enzymatic assays, since the motif DXSDE present in LDL domains is involved in calcium binding. Nevertheless, the functional aspects of these domains still need further investigation. In the tick *Haemaphysalis longicornis*, a similar serine peptidase (HlSP) which contains the CUB domain, was characterized. This enzyme is also up-regulated during feeding, is capable of albumin hydrolysis and presents an optimum pH of 5 against synthetic substrates [76]. In contrast to the acidic characteristics of HlSP, using scorpion MMG samples, it was not possible to observe Z-FR-MCA hydrolysis at pHs below 7 in the absence of reducing agents. Such difference in the pH of action could be related to distinct feeding habits or distinct organization/compartmentalization of the digestive tract.

Astacins, as TsCLTSP3, are not abundant but they likely also perform a role in the digestive process extracellularly. This comes from the observation that the digestive juice of the spider *Argiope aurantia* contains astacin-like enzymes [40]. Moreover, in our group using the same approach of this work we have identified 26 different astacins in the digestive juice of the spider *Nephilengys cruentata* (Fuzita et al., unpublished results).

### Evolutionary aspects of digestive peptidases

Scorpions have diverged from other arachnids at least 428 Ma [1]. Our analysis of specific DNA sequences allow us to make some evolutionary considerations on the digestive process in Arthropoda, more specifically in scorpions, but sometimes also in Metazoa in general. Two digestive strategies are observed among Metazoa: intracellular and extracellular digestion. Sometimes, both strategies are combined in the same organism and the extracellular digestion could be maximally represented by an extra-oral digestion. Intracellular digestion is a common feature in most invertebrate phyla and also in basal chordates. The taxa Placozoa, Porifera, Lophophorata [77] and the non-vertebrate chordates [78] rely exclusively on intracellular digestion. Platyhelminthes, Nemertea, Annelida, Mollusca [77], Chelicerata [79] and Crustacea [80] perform both intra and extracellular digestion. Ctenophora, Onycophora, Tardigrada [77], Myriapoda [81,82], Hexapoda [61] and Vertebrata [78] digest the food primarily extracellularly. Thus, in general, intracellular digestion is associated with a less-structured digestive system which is found mainly in basal groups.

Several common molecular features are obvious in well-studied groups with intracellular digestion. In these animals the “acidic proteolytic cocktail” (APC), composed by cysteine peptidases such as legumain, cathepsins B and L and the aspartic peptidase cathepsin D, plays an important role in food digestion intracellularly, not precluding its use extracellularly nor the use of serine peptidases from the trypsin family and also astacin-like metallopeptidases. This is observed in arachnids such as scorpions (this study), spiders (Fuzita et al., unpublished results) and ticks [37,38]; in crustaceans [83–86]; platyhelminths [87] and mollusks [88,89]. Moreover, the ACP genes are present in the ancient Placozoa lineage (*Trichoplax adhaerens*), evincing its early ancestry prior to the appearance of Eumetazoa.

Due to the need of a reducing environment for cysteine peptidase activity, it is parsimonious to assume that the initial use of ACP was targeted at food digestion intracellularly, inside membranous structures. Thus, serine peptidase would be a “best choice” for a proteolytic digestion, which has to be functional in a more oxidative environment. However, in some specific situations, the typical lysosomal cysteine peptidases are secreted. This is the case in some suborders of Coleoptera and Hemiptera [61]. The analysis of the evolution of these particular genes could give us some clues of the use and evolution of ACP.

#### Cathepsin L

Cathepsin L is a ubiquitous cysteine endopeptidase, indicating an early ancestral origin in life forms [90]. In humans, it is a common lysosomal enzyme [48] but it can be secreted under abnormal conditions such as in tumors [91]. In invertebrates, CTSL can be used for food digestion intracellularly as observed in ticks [38] and crustaceans [84] but it can also be secreted for luminal digestion [87,92]. In the present work it was shown that cathepsin L plays an important role in food protein digestion in the MMG of the scorpion *Tityus serrulatus*. As scorpions are ancient extant arthropods, a phylogenetic tree was constructed using CTSL sequences available from public databases, including the referred taxon (S8 Fig).

All Arachnida cathepsins L grouped together with the only exception of Parasitiformes and two Opiliones sequences which grouped with the parasitiforms. However, this result is consistent with the recent arthropod molecular phylogeny in which Opiliones is sister group to Parasitiformes + Pseudoscorpiones [93]. The divergence of the Parasitiformes sequences may be associated to specific selective pressures for specialized blood digestion. We could detect at least four gene duplications leading to different arachnid paralogs. The data evinces the importance of CTSL in food digestion, since phylogenetic analyses indicate that this gene was already duplicated in the arachnid ancestor, and it kept duplicating even after its divergence (Fig 7A and S8 Fig).

#### Legumain

Legumain is a cysteine endopeptidase of the C13 family (clan CD) with preference for asparagine residues in P1 position [90]. Although it was first identified in plants in the early 1980s [94], only in the last years this enzyme was identified and characterized in a large variety of metazoans. In different invertebrate groups legumain is associated with food digestion, usually under acidic conditions. This has already been shown in Nematoda [95], Platyhelminthes [87,96], Cephalochordata [97] and Arachnida-Parasitiformes [37,52].

In *Tityus serrulatus* this enzyme also seems to be related to the feeding stimulus once it was identified at both mRNA and proteomic levels only in fed animals. Due to the particular sequence of LEG, which lacks the C-terminal extension, a phylogenetic tree was constructed. However, the LEG phylogenetic position was not clear and it only grouped with Araneae sequences with a low posterior probability (S9 Fig). Also using the maximum likelihood algorithm they did not group together (data not shown). Recently, the legumain structure was determined and it was shown that the C-terminal extension has a death domain-like fold [98]. This domain is important for stabilization in pHs above 6 after legumain activation by trypsin or after interaction with integrins. We hypothesize that this stability in pHs above 6 could explain how legumain is used outside the lysosomes. Other arachnid legumain sequences from ticks and spiders also have the C-terminal extension which leads us to believe that the lack of this domain is exclusive to scorpions.

Albeit TsLEG did not provide substantial information about legumain evolution, an interesting evolutionary aspect was observed prior to the phylogenetic analysis. Although limited data are available, it seems that this enzyme is important to animals which have liquid/liquefied diets. This is true for the above cited references and also *Tetranychus urticae*, a mite (Acariformes) which feeds from plant sap, possesses the largest number of different legumain sequences deposited in public databases (S9 Fig). Moreover, in most insect orders (even the ones with complete genomes like Diptera, Coleoptera, Hymenoptera, Phtiraptera and Lepidoptera), a legumain gene is lacking, with the exception of hemipterans that are sap or blood feeders (S9 Fig). The hemipteran *Dysdercus peruvianus* also has at least 3 legumain genes which are still not available in public databases (Terra W.R., personal communication). It is more parsimonious to assume that the ancestor insect lost the legumain gene with a subsequent acquisition by hemipterans probably stimulated by the feeding habit from the ancestor. So far, this is empirical evolutionary evidence which requires further investigation.

## Conclusions

A combination of high-throughput sequence analytical techniques with an enzymological approach was applied for the first time to study the molecular physiology of digestion in a scorpion. Endo- and exopeptidases, carbohydrases and lipases were transcriptomically and proteomically identified. The enzymological assays allowed the inference of zymogens from cysteine peptidases activated under acidic conditions and also that acidic initial protein digestion, which is mainly performed by cathepsin L, seems to be quantitatively more important in contrast to the alkaline one. These results were further confirmed by quantitative mass spectrometry. Based on our data, the most complete molecular mechanism of digestion in the scorpion *Tityus serrulatus* can be proposed. The secretory granules are ready for the next predation event in the MMG of fasting animals. Some of the proteins involved in extracellular digestion (e.g. chitinases) are more represented in fasting animals whereas the ones involved in intracellular digestion are more abundant in fed animals (e.g. cathepsin L1). A chitolectin (chitinase 3) with a peritrophin domain that possibly is involved in the formation of a peritrophic gel/ membrane was, for the first time, identified in a scorpion. Evolutionarily, scorpions use a proteolytic cocktail similar to other animals which rely on intracellular digestion, and at least four cathepsin L gene duplications occurred in the arachnid ancestor, which kept duplicating after divergence of their lineages. The availability of these protein sequences opens the doors for future research of the digestive process dynamics and the use of recombinant enzymes including the preparation of antibodies for *in situ* location. Furthermore, the generated data about the physiology of digestion in *Tityus serrulatus* is very informative for the future development of scorpion specific control strategies.

## Supporting Information

S1 Fig Gene ontology terms of biological process, molecular function and cellular component from transcriptomic data. (TIF)

S2 Fig Gene ontology terms of biological process, molecular function and cellular component from proteomic data. (TIF)

S3 Fig Relative quantification of the possible digestive enzymes by mass spectrometry. Data from S4 Table were used for relative quantification of digestive enzymes abundance.
(TIF)

S4 Fig Gel filtration fractionation of crude MMG homogenate from *Tityus serrulatus*. Superdex G75 column was equilibrated with 20 mM Tris-HCl buffer (pH 7.0). Activated (●) or non-activated (○) fractions were assayed using different endopeptidase substrates to determine presence of zymogens. (A) Z-FR-MCA, pH 3; (B) hemoglobin, pH 2.8. Buffers used: 0.1 M citrate-phosphate containing 3 mM cysteine and 3 mM EDTA.
(TIF)

S5 Fig Effect of pH on stability of cysteine peptidases present in MMG crude homogenate samples. Samples were incubated at 30°C for 3 h (●) or at -20°C for 24 h (○). Buffers used (50 mM): pHs 2.6–7, citrate phosphate; pH 7.5–9 Tris-HCl. All buffers contained 3 mM cysteine and 3mM EDTA.
(TIF)

S6 Fig Purification of two cysteine peptidases from *Tityus serrulatus* MMG. A) Schematic representation of purification steps. (B) Chromatography of supernatant from ammonium sulfate fractionation on a HiTrap Butyl column equilibrated in 50 mM phosphate buffer (pH 6.0). Samples were eluted using a gradient of 1.7–0 M ammonium sulfate in same buffer. (C) Chromatography of active fractions from previous chromatography step (after desalting), represented by open circles (○), on a Resource S column equilibrated with 50 mM citrate-phosphate buffer (pH 5.0) (C1). Samples were eluted in gradient of 0–0.6 M sodium chloride in same buffer. (D) SDS-PAGE of samples exhibiting maximal activity, generated after cation-exchange chromatography, represented by open squares (□). Substrate used to follow activity in all steps was 10 **μ**M Z-FR-MCA in 0.1 M citrate-phosphate buffer (pH 5.5) containing 3 mM cysteine and 3 mM EDTA. MMTS was added to final concentration of 1 mM to fractions exhibiting activity. S, standard (kDa).
(TIF)

S7 Fig Cysp1 inhibition by pepstatin. (A) Lineweaver-Burk plots obtained with different pepstatin concentrations [Control (□); 1 μM (○), 5 μM (■), 10 μM (*) pepstatin]. Assays were performed using purified cysp1 in 0.1 M citrate-phosphate buffer (pH 5.5) with Z-FR-MCA. (B) Replot of the slopes of curves obtained from Lineweaver-Burk plots against pepstatin concentration, indicating a K_i_ value of 40 Ass
(TIF)

S8 Fig Cathepsin-L phylogeny using maximum likelihood algorithm. Blue circles display high bootstrap values showing closest branches to tips having ≥ 0.95 support, indicating that all four duplications are supported when more inclusive clades are considered. Accession numbers are shown in the figure together with taxa names. The sequences from *Nephilengys cruentata* and *Neosadocus sp* are from our unpublished results and are not yet available in public databases. Sequences from *Dysdercus peruvianus* were kindly forgiven by Dr. Walter Terra from the Chemistry Department of Universidade de Srom rt, indicating that al000 cycles.
(TIF)

S9 Fig Bayesian phylogeny of LEG (including C-terminal region) + GPIt. Clade posterior probabilities are shown. GPIt and LEG sequences separate into reciprocally monophyletic clades. Note that *T*. *serrulatus* appears in Arachnida with high support for GPIt, but its LEG sequence has low support (due to larger divergence).
(TIF)

S1 Table Assay conditions and methods used in determination of peptidase activities from *Tityus serrulatus* midgut and midgut glands. (DOCX)

S2 Table Summary of *de novo* assembly results. (XLSX)

S3 Table Identified proteins by shotgun proteomics in MMG of *Tityus serrulatus*. (XLSX)

S4 Table Possible digestive enzymes identified by proteomics analysis. Mass spectrometry data are from 3 different biological samples. Percentage of normalized spectra counting (NSC) is shown as quantitative value to each enzyme. In the middle a prediction of the subcellular location using WoLF PSORT and in the right the presence of GO term related to extracellular space and lysosome is displayed. *k*-NN, *k*-nearest neighbor classifier from PSORT; Ex, extracellular space; Ly, lysosome; E.R., endoplasmatic reticulum; Cy, cytosol; Mi, mitochondria; Nu, nucleus; Pe, peroxisome, Pl, plasma membrane. N.I, not identified. *the protein was not identified in all triplicate samples. **sequences with incomplete N-terminal region.—no result.—not measured.
(XLSX)

S5 Table Purification of cysteine endopeptidases from *Tityus serrulatus´* MMG. Substrate used was 10 **μ**M Z-FR-MCA diluted in 0.1M citrate-phosphate buffer containing 3.0 mM cysteine and 3.0 mM EDTA.
(XLSX)

S6 Table Proteins identified by mass spectrometry in C1 activity pool. (XLSX)

S7 Table Kinetic parameters of purified cysp1 and cysp 2 using two different substrates. Kinetic parameters (means and S.E.M.) were determined using Enzfitter.
(XLSX)

S1 Dataset Assemblies from fed and fasting scorpions transcriptome. (RAR)

S2 Dataset Amino acid database used for protein identification. (FASTA)

S3 Dataset Peptide report from entire proteome data. (XLSX)

S4 Dataset CA-074 inhibition. (PDF)
