# Supplementary figures and images for: Biochemical, Transcriptomic and Proteomic Analyses of Digestion in the Scorpion Tityus serrulatus: Insights into Function and Evolution of Digestion in an Ancient Arthropod
Source: PLoS One. 2015 Apr 15;10(4):e0123841. doi: 10.1371/journal.pone.0123841 (PMC4398375; doi:10.1371/journal.pone.0123841)

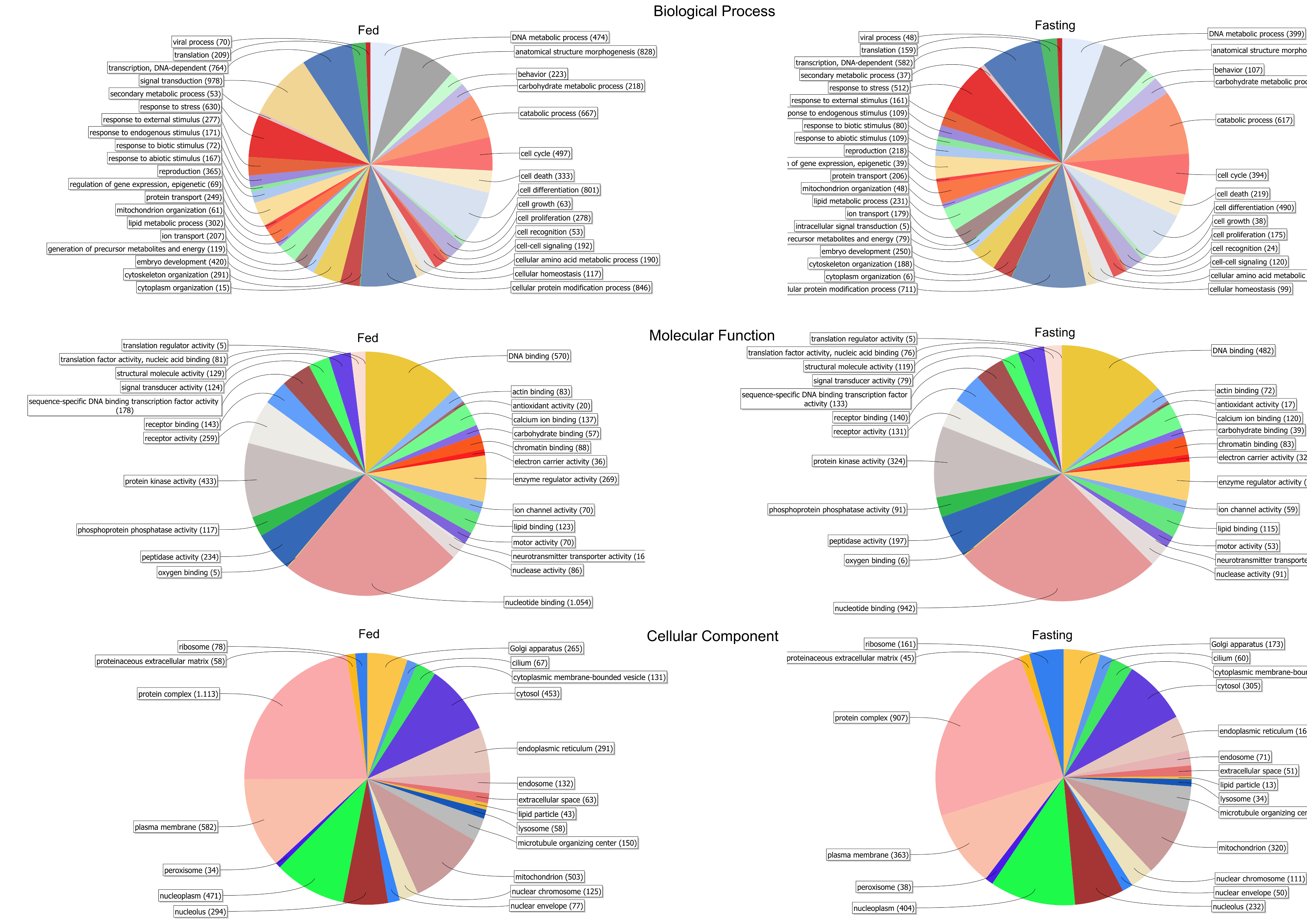

Supplement: S1 Fig — (TIF) [file pone.0123841.s001.tif]

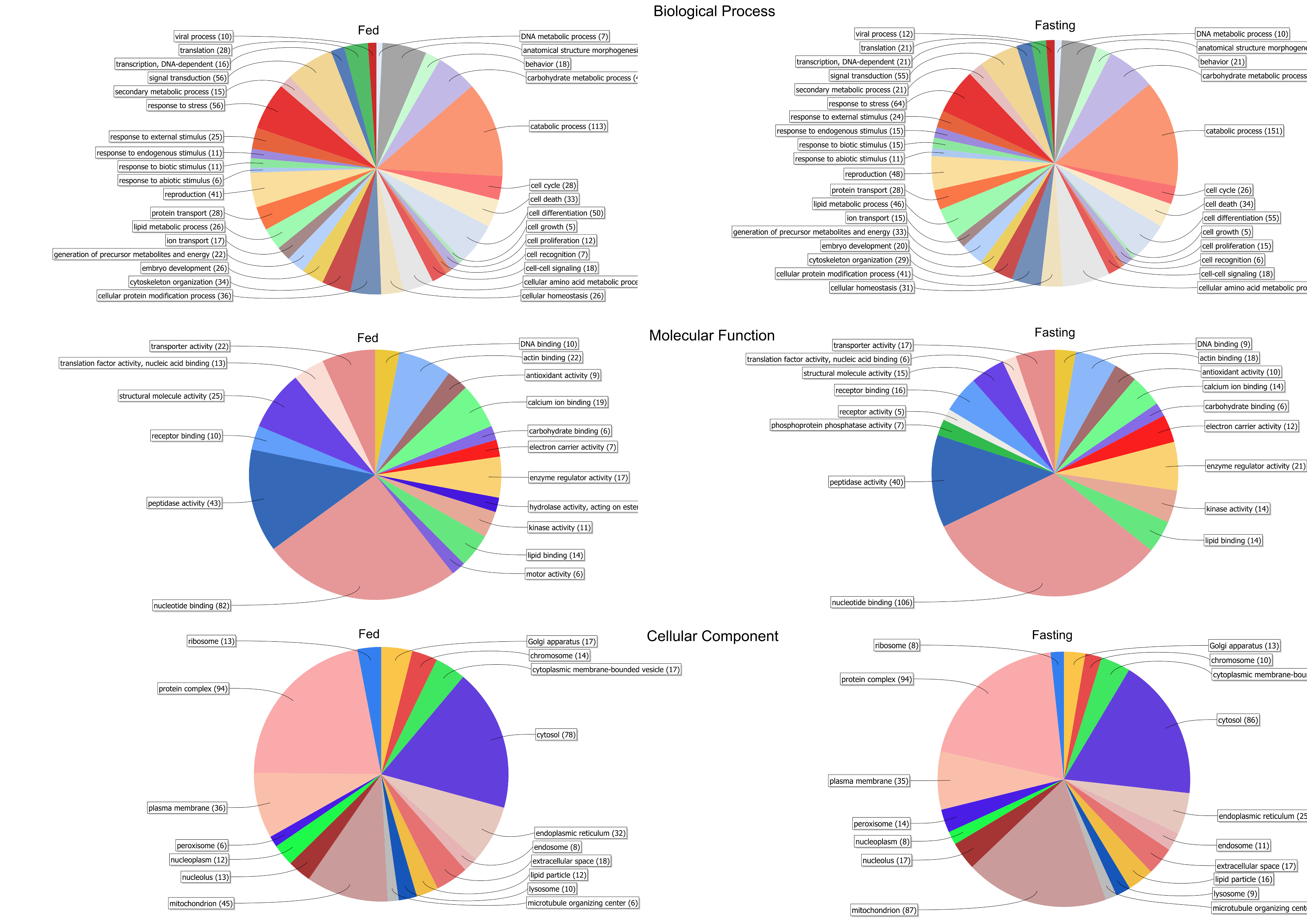

Supplement: S2 Fig — (TIF) [file pone.0123841.s002.tif]

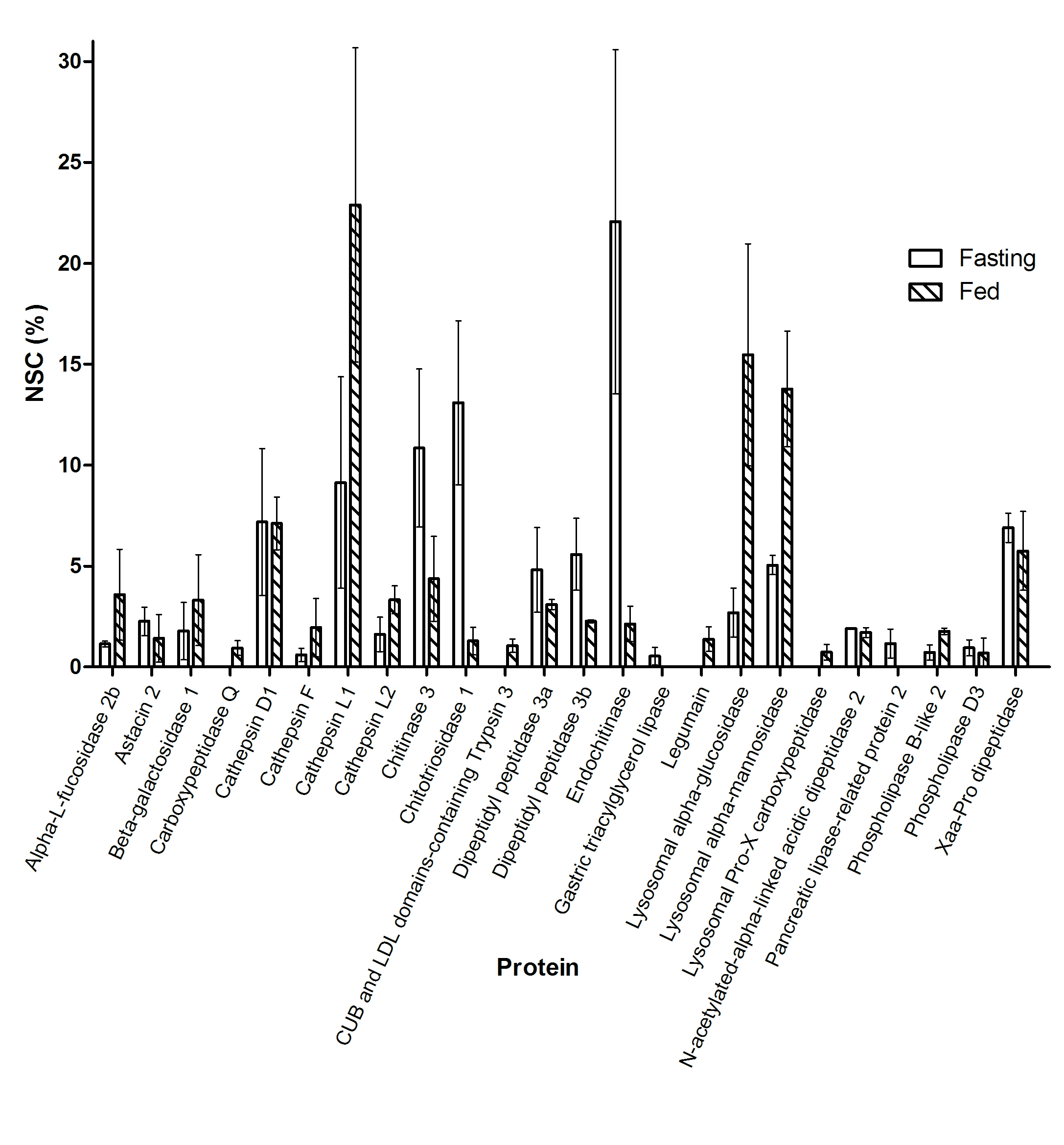

Supplement: S3 Fig — Data from S4 Table were used for relative quantification of digestive enzymes abundance. (TIF) [file pone.0123841.s003.tif]

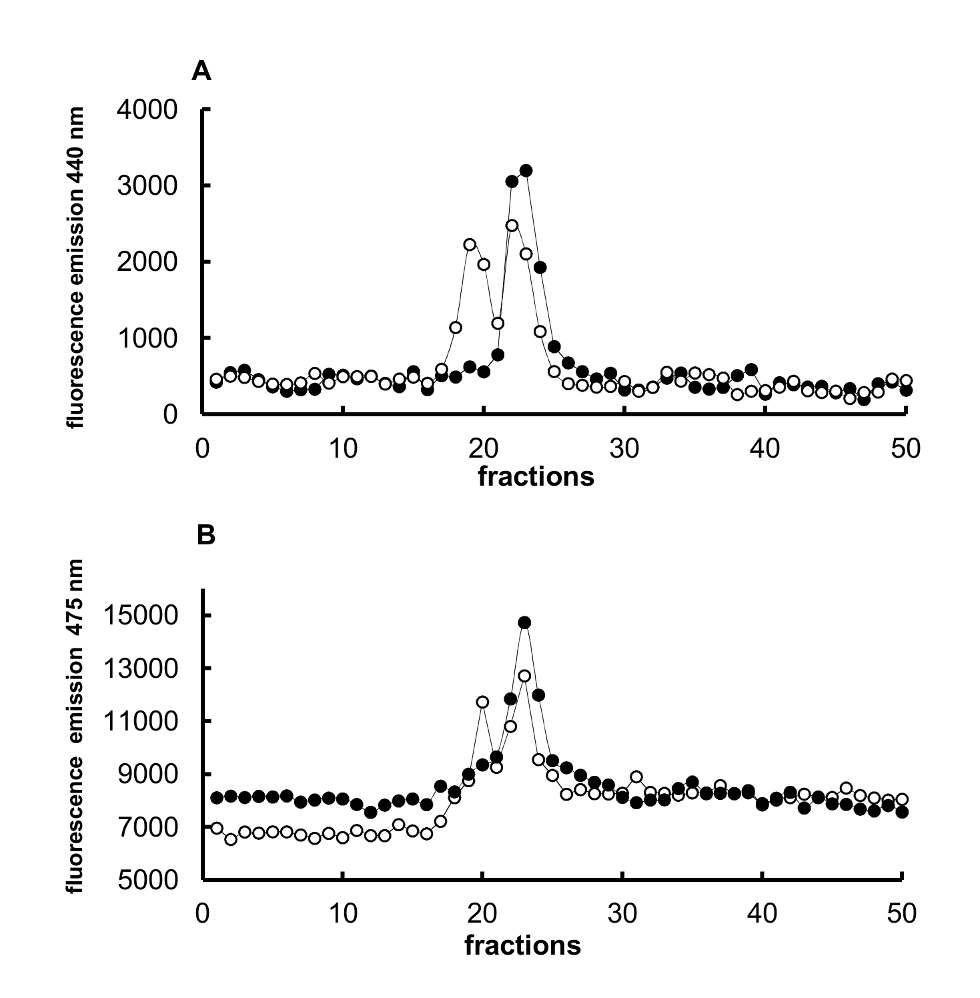

Supplement: S4 Fig — Superdex G75 column was equilibrated with 20 mM Tris-HCl buffer (pH 7.0). Activated (●) or non-activated (○) fractions were assayed using different endopeptidase substrates to determine presence of zymogens. (A) Z-FR-MCA, pH 3; (B) hemoglobin, pH 2.8. Buffers used: 0.1 M citrate-phosphate containing 3 mM cysteine and 3 mM EDTA. (TIF) [file pone.0123841.s004.tif]

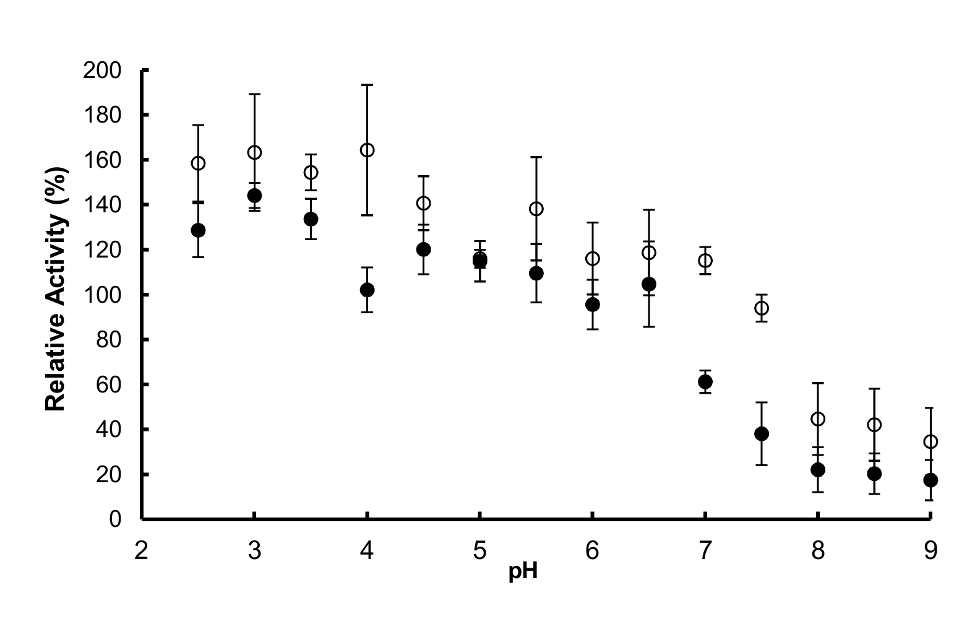

Supplement: S5 Fig — Samples were incubated at 30°C for 3 h (●) or at -20°C for 24 h (○). Buffers used (50 mM): pHs 2.6–7, citrate phosphate; pH 7.5–9 Tris-HCl. All buffers contained 3 mM cysteine and 3mM EDTA. (TIF) [file pone.0123841.s005.tif]

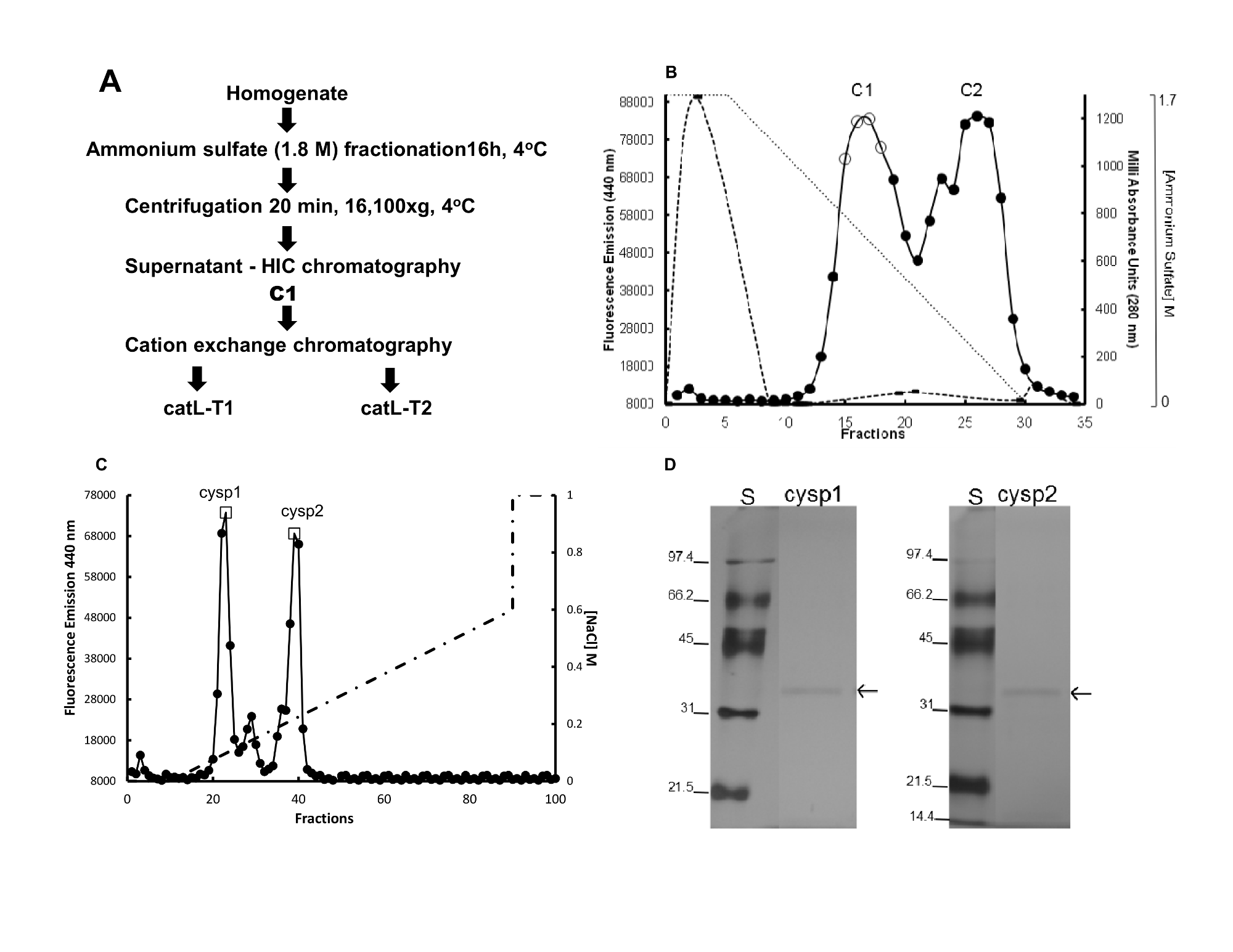

Supplement: S6 Fig — A) Schematic representation of purification steps. (B) Chromatography of supernatant from ammonium sulfate fractionation on a HiTrap Butyl column equilibrated in 50 mM phosphate buffer (pH 6.0). Samples were eluted using a gradient of 1.7–0 M ammonium sulfate in same buffer. (C) Chromatography of active fractions from previous chromatography step (after desalting), represented by open circles (○), on a Resource S column equilibrated with 50 mM citrate-phosphate buffer (pH 5.0) (C1). Samples were eluted in gradient of 0–0.6 M sodium chloride in same buffer. (D) SDS-PAGE of samples exhibiting maximal activity, generated after cation-exchange chromatography, represented by open squares (□). Substrate used to follow activity in all steps was 10 μM Z-FR-MCA in 0.1 M citrate-phosphate buffer (pH 5.5) containing 3 mM cysteine and 3 mM EDTA. MMTS was added to final concentration of 1 mM to fractions exhibiting activity. S, standard (kDa). (TIF) [file pone.0123841.s006.tif]

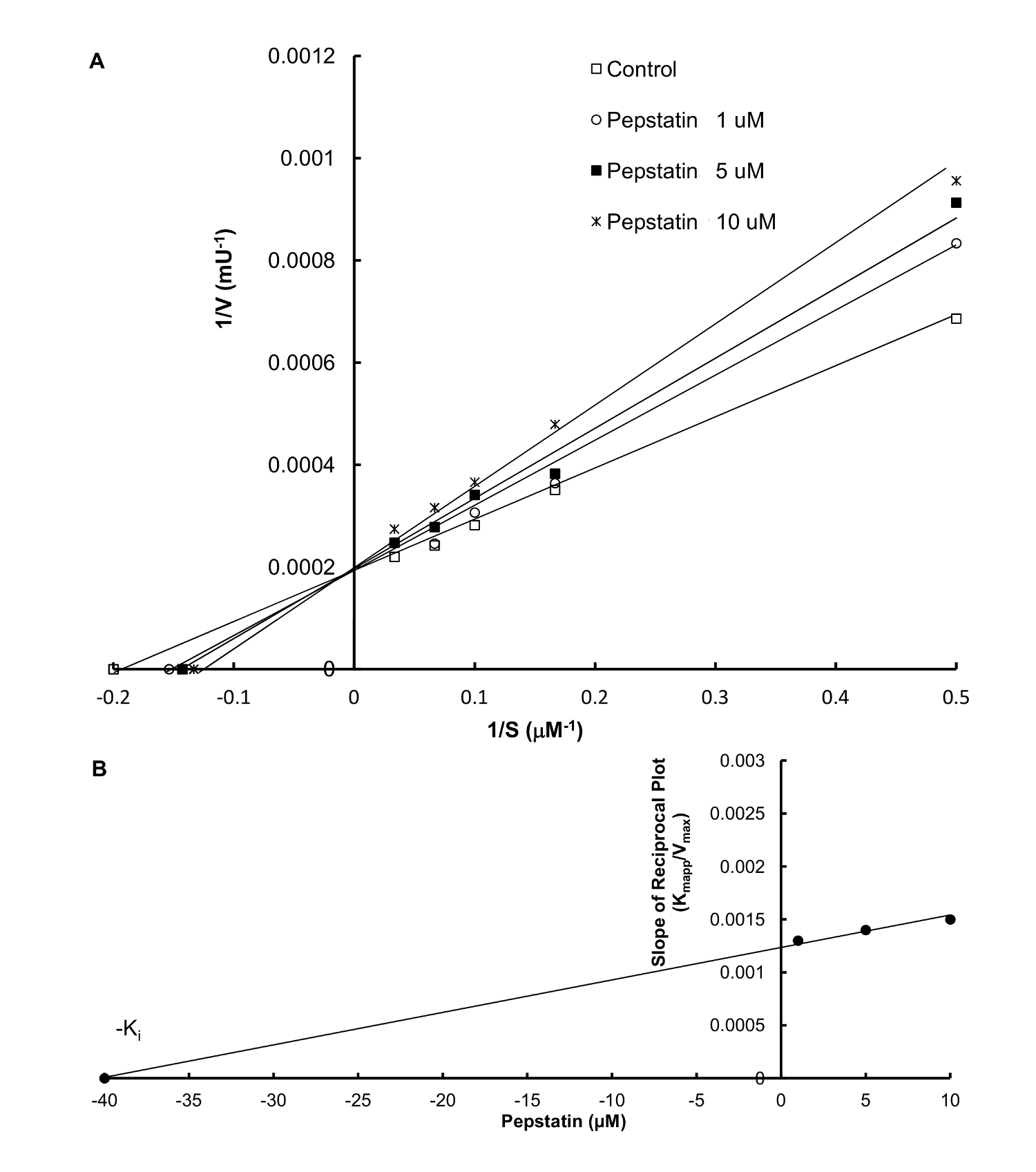

Supplement: S7 Fig — (A) Lineweaver-Burk plots obtained with different pepstatin concentrations [Control (□); 1 μM (○), 5 μM (■), 10 μM (*) pepstatin]. Assays were performed using purified cysp1 in 0.1 M citrate-phosphate buffer (pH 5.5) with Z-FR-MCA. (B) Replot of the slopes of curves obtained from Lineweaver-Burk plots against pepstatin concentration, indicating a Ki value of 40 Ass (TIF) [file pone.0123841.s007.tif]

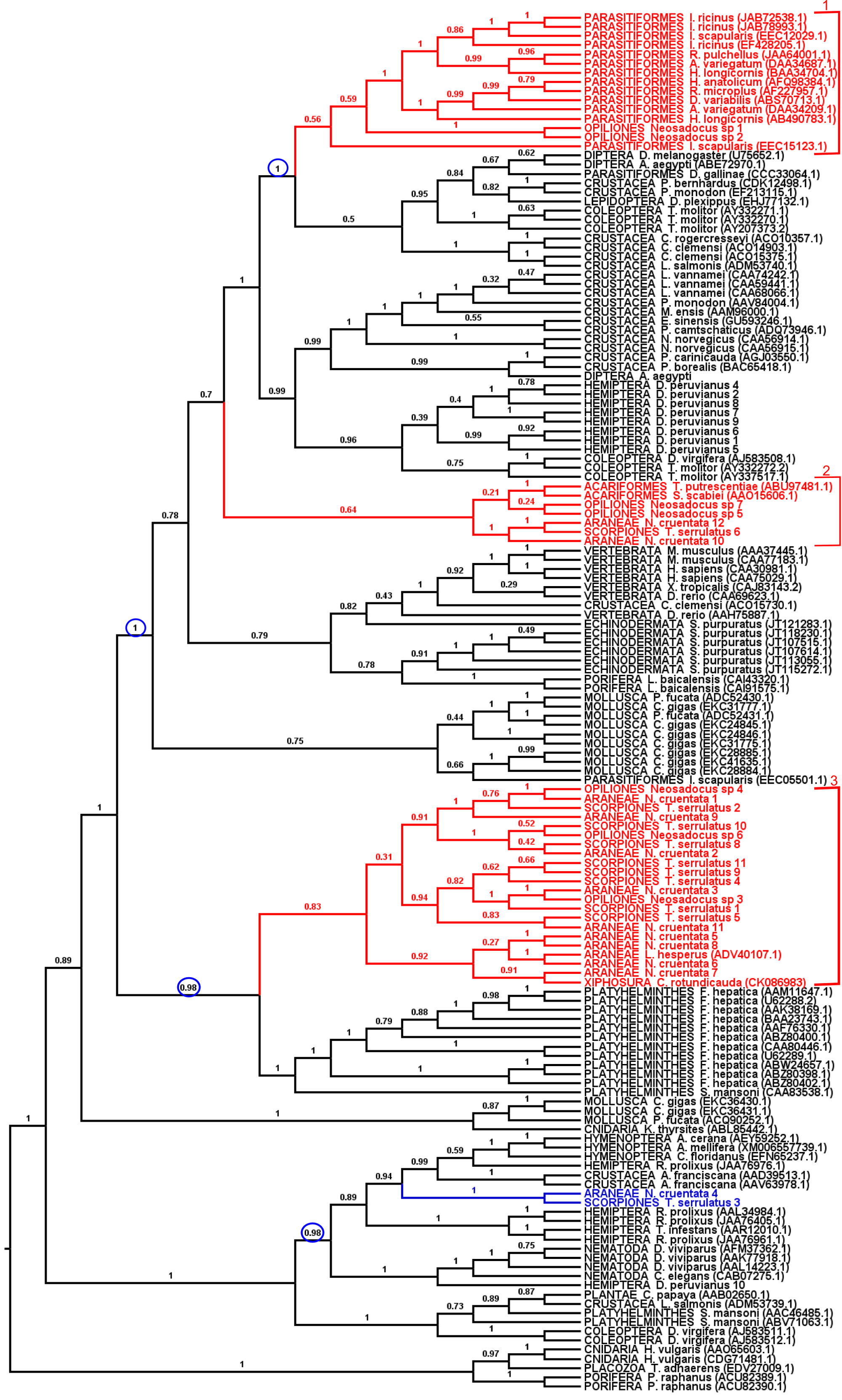

Supplement: S8 Fig — Blue circles display high bootstrap values showing closest branches to tips having ≥ 0.95 support, indicating that all four duplications are supported when more inclusive clades are considered. Accession numbers are shown in the figure together with taxa names. The sequences from Nephilengys cruentata and Neosadocus sp are from our unpublished results and are not yet available in public databases. Sequences from Dysdercus peruvianus were kindly forgiven by Dr. Walter Terra from the Chemistry Department of Universidade de Srom rt, indicating that al000 cycles. (TIF) [file pone.0123841.s008.tif]

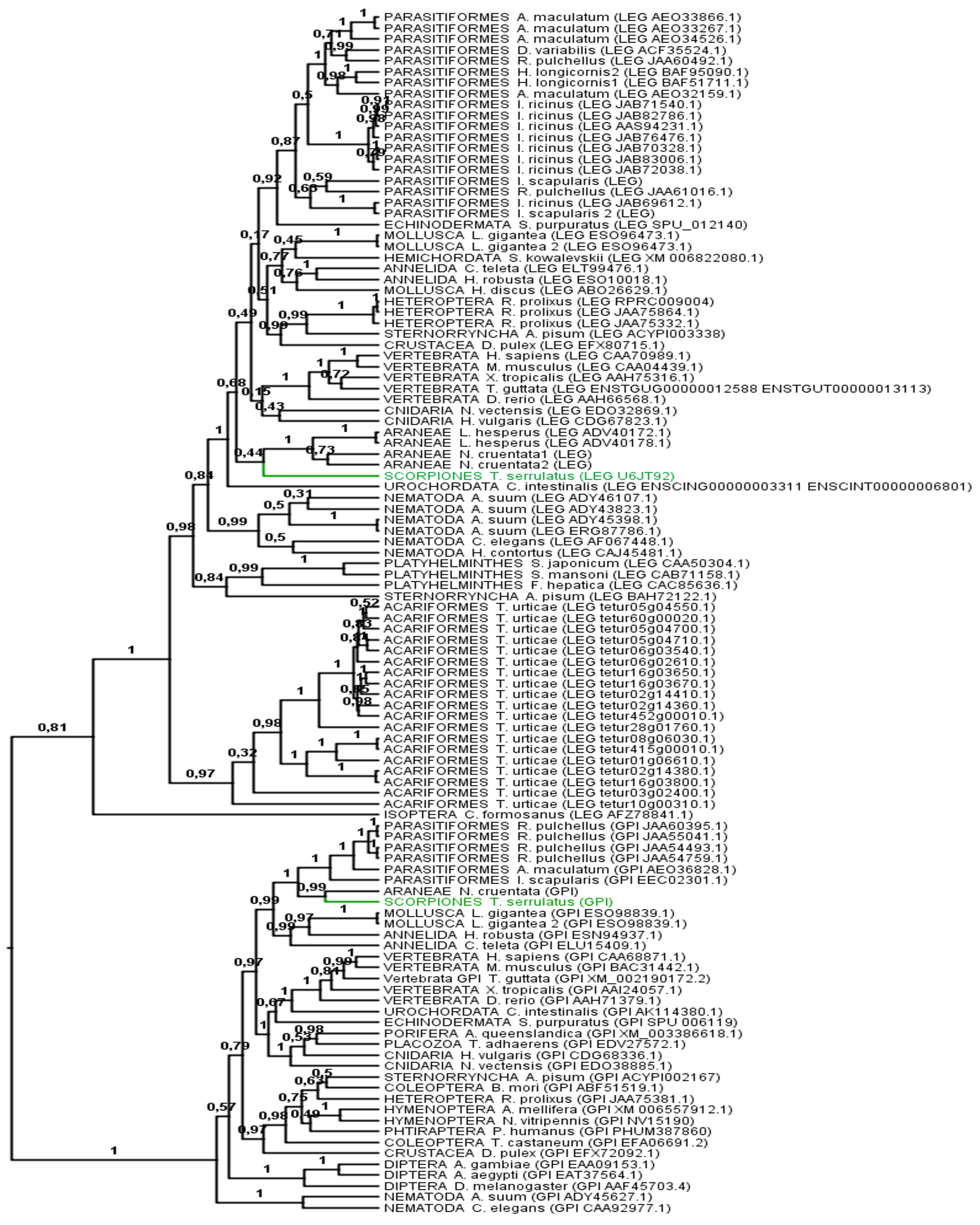

Supplement: S9 Fig — Clade posterior probabilities are shown. GPIt and LEG sequences separate into reciprocally monophyletic clades. Note that T. serrulatus appears in Arachnida with high support for GPIt, but its LEG sequence has low support (due to larger divergence). (TIF) [file pone.0123841.s009.tif]
