# Supplementary material for: Biochemical, Transcriptomic and Proteomic Analyses of Digestion in the Scorpion Tityus serrulatus: Insights into Function and Evolution of Digestion in an Ancient Arthropod
Source: PLoS One. 2015 Apr 15;10(4):e0123841. doi: 10.1371/journal.pone.0123841 (PMC4398375; doi:10.1371/journal.pone.0123841)
Supplement: S1 Table — (DOCX) [file pone.0123841.s010.docx]

| **Enzyme Class** | **Enzyme** | **Substrate** | **Buffer (mM); pH** | **Reference** |
| --- | --- | --- | --- | --- |
| **Cysteine peptidase*** | **Cathepsin L** | Z-FR-MCA; 10 µM | Citrate-phosphate 100; 2.6-6  MES 100; 6-7  TRIS-HCl 100; 7-9 | Lemos and Terra, 1991;  Sogawa and Takahashi,1978 |
|  |  | Hemoglobin; 2% | Gly-HCl 100; 1,5-2  Citrate-phosphate 100; 2,6-3,8 | Melo et al., 2001 |
|  |  | Abz-FRQ-EDDnp; 0.1 µM | Citrate-phosphate 100; 3, 5.5 | Cotrin et al., 2004 |
|  |  | Abz-GIVRAK-EDDnp; 0.1µM | Citrate-phosphate 100; 3, 5.5 |  |
|  |  | Abz-GIVRPK-EDDnp; 0.1µM | Citrate-phosphate 100; 3, 5.5 |  |
|  | **Cathepsin B** | Z-RR-MCA; 10µM | Citrate-phosphate 100; 3, 5.5 | Rawlings and Salvesen, 2012 |
|  |  | Abz-GIVRAK(Dnp)-OH; 0.1 µM | Citrate-phosphate 100; 3, 5.5 | Cotrin et al., 2004 |
|  |  | Abz-GIVRPK(Dnp)-OH; 0.1 µM | Citrate-phosphate 100; 3, 5.5 |  |
|  |  | Z-AAN-MCA; 30 µM | Citrate-phosphate 100; 3, 5.5 | Chen et al., 1997 |
|  |  | Z-VAN-MCA; 30 µM | Citrate-phosphate 100; 3, 5.5 |  |
| **Aspartic peptidase** | **Cathepsin D** | Hemoglobin; 2% | Gly-HCl 50; 1,5-2  Citrate-phosphate 100; 2,6-3,8 | Lemos and Terra, 1991;  Sogawa and Takahashi, 1978 |
|  |  | Abz-AIAFFSRQ-EDDnp; 0.1µM | Gly-HCl 100; 2.8 | Pimenta et al, 2001 |
| **Serine peptidase**** | **Trypsin** | Z-FR-MCA; 0.1µM | Citrate-phosphate 100; 2.6-6  MES 100; 6-7  TRIS-HCl 100; 7-9 | Rawlings and Salvesen, 2013 |
|  |  | Casein-FITC; 0.2 % | MES 100; 6-7  TRIS-HCl 100; 7-9  Gly-HCl 100; 9-10 | Twining , 1984 |
|  |  | Z-GGR-MCA; 10µM | TRIS-HCl 100; 8 | Lopes et al., 2003 |
|  | **Chymotrypsin** | N-Suc-AAPF-MCA; 10µM | TRIS-HCl 100; 8 | Lopes et al., 2009 |
|  |  | Casein-FITC; 0.2 % | MES 100; 6-7  TRIS-HCl 100; 7-9  Gly-HCl 100; 9-10 | Twining, 1984 |
| **Metallopeptidase** | **Astacin** | Casein-FITC; 0.2 % | MES 100; 6-7  TRIS-HCl 100; 7-9  Gly-HCl 100; 9-10 | Twining, 1984 |
|  |  | Abz-GPKRAPWV-EDDnp; 0.1µM | TRIS-HCl 100; 8.5 | -- |

*Cysteine peptidase assay buffers contain 3 mM cysteine and 3 mM EDTA

**Serine peptidase assay buffers contain 10 mM CaCl2

Excitation/emission wavelengths: MCA 340 nm/440 nm ; EDDnp, Dnp 320 nm/420 nm; FITC 365 nm/525 nm; Fluorescamine 375 nm/475 nm

**Complementary references**

Alves LC, Almeida PC, Franzoni L, Juliano L, Juliano MA. Synthesis of Nα-protected aminoacyl 7-amino-4-methyl-coumarin amide by phosphorous oxychloride and preparation of specific fluorogenic substrates for papain. Peptide Research. 1996; 9: 92–96.

Chen JM, Dando PM, Rawlings ND, Brown MA, Young NE, Stevens RA, et al. Cloning, isolation, and characterization of mammalian legumain, an asparaginyl endopeptidase. Journal of Biological Chemistry. 1997;272(12):8090-8.

Cotrin SS, Puzer L, Judice WAD, Juliano L, Carmona AK, Juliano MA. Positional-scanning combinatorial libraries of fluorescence resonance energy transfer peptides to define substrate specificity of carboxydipeptidases: assays with human cathepsin B. Analytical Biochemistry. 2004;335(2):244-52.

Lemos FJA, Terra WR. PROPERTIES AND INTRACELLULAR-DISTRIBUTION OF A CATHEPSIN-D-LIKE PROTEINASE ACTIVE AT THE ACID REGION OF MUSCA-DOMESTICA MIDGUT. Insect Biochemistry. 1991;21(5):457-65.

Lopes AR, Terra WR. Purification, properties and substrate specificity of a digestive trypsin from Periplaneta americana (Dictyoptera) adults. Insect Biochemistry and Molecular Biology. 2003;33(4):407-15.

Lopes AR, Saro PM, Terra WR. INSECT CHYMOTRYPSINS: CHLOROMETHYL KETONE INACTIVATION AND SUBSTRATE SPECIFICITY RELATIVE TO POSSIBLE COEVOLUTIONAL ADAPTATION OF INSECTS AND PLANTS. Archives of Insect Biochemistry and Physiology. 2009;70(3):188-203. doi: 10.1002/arch.20289.

Melo RL, Alves LC, Del Nery E, Juliano L, Juliano MA. Synthesis and hydrolysis by cysteine and serine proteases of short internally quenched fluorogenic peptides. Analytical Biochemistry. 2001;293(1):71-7. doi: 10.1006/abio.2001.5115.

Sogawa K, Takahashi K. USE OF FLUORESCAMINE-LABELED CASEIN AS A SUBSTRATE FOR ASSAY OF PROTEINASES. Journal of Biochemistry. 1978;83(6):1783-7. PubMed PMID: WOS:A1978FA86200032.

Twining SS. FLUORESCEIN ISOTHIOCYANATE-LABELED CASEIN ASSAY FOR PROTEOLYTIC-ENZYMES. Analytical Biochemistry. 1984;143(1):30-4.
