# Supplementary material for: Biochemical, Transcriptomic and Proteomic Analyses of Digestion in the Scorpion Tityus serrulatus: Insights into Function and Evolution of Digestion in an Ancient Arthropod
Source: PLoS One. 2015 Apr 15;10(4):e0123841. doi: 10.1371/journal.pone.0123841 (PMC4398375; doi:10.1371/journal.pone.0123841)
Supplement: S4 Dataset — (PDF) [file pone.0123841.s020.pdf]

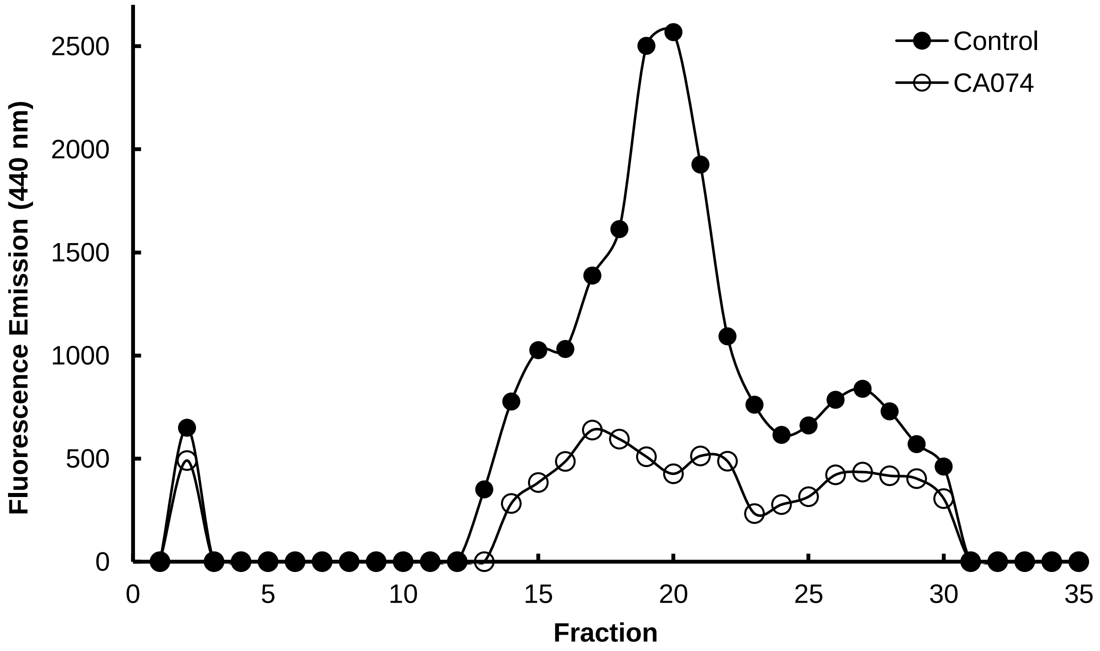

Supplementary data 4: CA-074 (10 μM) inhibition in hydrophobic chromatographic fractioning of *Tityus serrulatus* MMG homogenate. Inhibitory and activity assays were performed as described in the methodolgy. Activity was measured using 10 μM Z-FR-MCA in 0.1 M citrate-phosphate buffer pH 5.5.
